# Intergenic Locations of Rice Centromeric Chromatin

**DOI:** 10.1371/journal.pbio.0060286

**Published:** 2008-11-25

**Authors:** Huihuang Yan, Paul B Talbert, Hye-Ran Lee, Jamie Jett, Steven Henikoff, Feng Chen, Jiming Jiang

**Affiliations:** 1 Department of Horticulture, University of Wisconsin-Madison, Madison, Wisconsin, United States of America; 2 Howard Hughes Medical Institute, Fred Hutchinson Cancer Research Center, Seattle, Washington, United States of America; 3 United States Department of Energy Joint Genome Institute, Walnut Creek, California, United States of America; University of Missouri-Columbia, United States of America

## Abstract

Centromeres are sites for assembly of the chromosomal structures that mediate faithful segregation at mitosis and meiosis. Plant and animal centromeres are typically located in megabase-sized arrays of tandem satellite repeats, making their precise mapping difficult. However, some rice centromeres are largely embedded in nonsatellite DNA, providing an excellent model to study centromere structure and evolution. We used chromatin immunoprecipitation and 454 sequencing to define the boundaries of nine of the 12 centromeres of rice. Centromere regions from chromosomes 8 and 9 were found to share synteny, most likely reflecting an ancient genome duplication. For four centromeres, we mapped discrete subdomains of binding by the centromeric histone variant CENH3. These subdomains were depleted in both intact and nonfunctional genes relative to interspersed subdomains lacking CENH3. The intergenic location of rice centromeric chromatin resembles the situation for human neocentromeres and supports a model of the evolution of centromeres from gene-poor regions.

## Author Summary

Before a cell divides, its chromosomes must be duplicated and then separated to provide each daughter cell with an identical genome copy. To accomplish this separation, the cell-division apparatus attaches to structures on the chromosomes called centromeres. Most plant and animal centromeres contain highly repetitive DNA sequences and specific proteins such as CENH3; however, it is not known which of the many repeats bind CENH3. Some rice centromeres, however, consist largely of single-copy DNA, providing a tractable model for investigating CENH3-binding patterns. Using modern DNA sequencing technology and an antibody to CENH3, we were able to find which sequences in the rice genome are bound by CENH3. We uncovered evidence that one centromere, *Cen8*, which has lost much of its repetitive content through a rearrangement within the last approximately 5 million years, is derived from a highly repetitive centromeric region that was duplicated along with the rest of the genome 50–70 million years ago. We also found that CENH3 is bound discontinuously in centromeric subdomains that have fewer genes than subdomains lacking CENH3. These results suggest, not only that centromeres evolve in gene-poor regions, but also how centromeres might evolve from single-copy to repetitive sequences.

## Abstract 3

A key centromere protein is found to bind discontinuously to subdomains of centromeres that are depleted in genes, suggesting that centromeres evolve in gene-poor regions.

## Introduction

Centromeres are the essential sites on eukaryotic chromosomes that assemble kinetochores for attachment to spindle microtubules in mitosis and meiosis. Multicellular eukaryotes have “regional” centromeres encompassing hundreds of kilobases that are often located in megabase-sized arrays of 150–180-bp AT-rich tandem repeats known as satellite DNA. Centromeric satellite repeats evolve rapidly and show little sequence conservation even between related species [1]. Despite the frequent occurrence of regional centromeres in satellite arrays, in many eukaryotes centromere location appears to be specified epigenetically [2] by the deposition of specialized nucleosomes containing a centromere-specific variant of histone H3, often known as CENH3 (Centromeric H3) or as CENP-A, after the mammalian centromeric histone [3]. Evidence for the epigenetic nature of centromeres comes from neocentromeres, the rare sites of spontaneous CENP-A nucleosome deposition described in humans, flies, and barley, that assemble functional kinetochores on DNA sequences that lack any sequence similarity to the normal centromeres [4,5]. Experimental overexpression of CENH3 in the fruit fly Drosophila melanogaster likewise produced functional kinetochores on normally noncentromeric sequences [6]. These observations suggest that CENH3 nucleosomes and kinetochores can assemble on any DNA sequence, but in nature, they normally prefer specific satellite sequences.

In animal centromeres, CENH3/CENP-A binding is discontinuous, with regions of CENH3/CENP-A nucleosomes interspersed with regions of H3-containing nucleosomes [7]. Similar discontinuous regions of CENP-A binding have been observed in human neocentromeres [8,9] and artificial chromosomes [10]. In rice (Oryza sativa), the binding domains for CENH3 (the National Center for Biotechnology Information [NCBI] Protein accession number AAR85315) have been mapped for two centromeres, *Cen3* and *Cen8* [11–13]. These two domains show evidence of containing both CENH3 and H3 nucleosomes, but previous methods were unable to map distinct CENH3-binding and CENH3-lacking subdomains. Precise mapping of CENH3 nucleosome subdomains is an essential first step in understanding both the structure of centromeres and why CENH3 nucleosomes are assembled and maintained at some locations and not others. Such precise mapping is not possible for most animal and plant centromeres because of highly repetitive satellite sequences, but some rice centromeres have relatively little of the rice-specific centromeric satellite sequence *CentO* [14]. Here, we use chromatin immunoprecipitation (ChIP) with 454 sequencing technology to map nine out of 12 rice centromeres, and to map CENH3-binding subdomains in the four centromeres that have the most complete sequence. We show that CENH3-binding subdomains are depleted in genes relative to adjacent CENH3-lacking subdomains, suggesting a model in which low transcriptional activity is important for the establishment of new centromere sequences and their evolution into mature centromeres.

## Results

### Physical and Sequence Maps of the Rice Centromeres

We previously defined the crossover-suppressed regions for two rice centromeres, *Cen3* [13] and *Cen8* [12], which span 3,113 kb and 2,312 kb, respectively. The CENH3-binding domains in these two centromeres were estimated to span approximately 1,810 kb and 750 kb, respectively, and reside within the crossover-suppressed regions. By using the same approach, we have defined the crossover-suppressed regions for the remaining ten centromeres and placed them on physical maps of the centromere regions (Figure S1). There were 14 physical gaps in the 12 rice centromeric regions of the current rice chromosome pseudomolecules (http://rice.plantbiology.msu.edu/). The gap in centromere 3 (*Cen3*) was previously estimated as approximately 450 kb based on fluorescent in situ hybridization (FISH) on extended DNA fibers [13]. The other 13 gaps had a combined size of approximately 5.9 Mb (Figure S1). Among them, the size for four gaps (∼0.54 Mb in total) was also estimated by FISH and fiber-FISH, including the 111-kb gap in *Cen4* [15], the 69-kb gap in *Cen10* [16], the 310-kb gap in *Cen7*, and the 50-kb in *Cen11* [17]. The remaining nine gaps, together approximately 5.35 Mb, were each sized by optical mapping [18]. Including these physical gaps, the sizes of the crossover-suppressed domains vary between 1,447 kb (*Cen10*) and 5,449 kb (*Cen6*).

Eight of the 12 centromeres have a *CentO*-containing sequence gap of at least 300 kb. In contrast, the remaining four centromeres contain only a limited amount of the *CentO* repeat (∼60–250 kb), including *Cen4* [15] and *Cen8* [11], whose *CentO* arrays have been fully sequenced, and *Cen5* and *Cen7*, which each have a <100-kb *CentO*-containing sequence gap. The DNA sequences of these four centromeres provide the foundation for using a high-throughput approach to profile the CENH3 occupancy in these centromeres.

### Boundaries of the CENH3-Binding Domains in Rice Centromeres

A rice anti-CENH3 antibody was used for ChIP, and the immunoprecipitated DNA was sequenced (ChIP-Seq) using the 454 sequencing technology. As expected, *CentO*-related reads were highly overrepresented in the 325,298 reads in this ChIP-Seq dataset, with 34.7% of the reads matching the *CentO* consensus for at least 20 bp (Figure S2). This represents over 17-fold enrichment genome-wide, considering that the approximately 7 Mb of *CentO* satellite repeats, as estimated by FISH and by optical mapping, nearly all of them present in the centromeric regions, account for less than 2% of the approximately 382-Mb rice genome [14,18].

**Figure 1:**
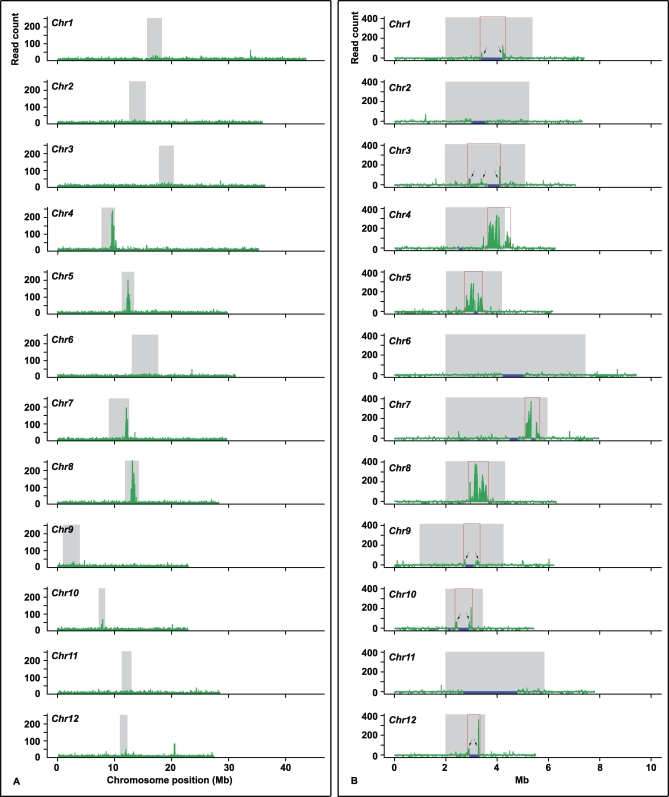
Plot of ChIP-Seq Sequence Reads along Individual Rice Chromosomes The region corresponding to the crossover-suppressed domain in each centromere is shadowed in gray. (A) The window size is 20 kb, spaced every 10 kb. Strong enrichment of sequence reads was observed in *Cen4*, *Cen5*, *Cen7*, and *Cen8*; less significant enrichment was also detected in *Cen10*. (B) For each centromere, 2 Mb of flanking sequence from each side of the crossover-suppressed domain was used as a control, except the short-arm side of *Cen9*, in which all of the 993-kb sequence was analyzed. The read density was calculated to represent the number of reads in each 20 kb based on the mappable sequence, as specified in Materials and Methods. The inferred boundary of the CENH3-binding domain is indicated by vertical red lines. In *Cen4*, the functional domain has extended approximately 100 kb into the flanking low-crossover domain at the long-arm side. Arrows indicate sequence read peaks that were selected for confirming enrichment by real-time PCR (see Figure S3 for details).

We used the BLAST program to map the remaining 212,086 *CentO*-less reads to the rice genome. To test our expectation that these mapped reads are highly enriched in the centromeric regions, we divided each chromosome into 20-kb windows, positioned every 10 kb, and plotted the read count within each window along its chromosome position. This analysis gave background reads on individual chromosome arms that appeared to be highly uniform, ranging from 7.4 to eight reads per 20 kb. These background reads most likely represent technical “noise” in the assay. However, we cannot exclude the possibility that they represent real noncentromeric signal, because CENH3 has been observed to localize at low levels throughout pachytene chromosomes in Arabidopsis thaliana [19] and is known to localize in euchromatin when overexpressed in animals [20]. The analysis also revealed conspicuous peaks within the four best-sequenced centromeres, spanning approximately 820 kb in *Cen4*, approximately 630 kb in *Cen5*, approximately 420 kb in *Cen7*, and approximately 815 kb in *Cen8* (Figure 1A). *Cen10* had a lower level of read enrichment; however, a region of approximately 610 kb that showed clear CENH3 binding was readily identified. In all five cases, the defined CENH3 domain spanned the entire *CentO*-containing sequence gap and the assembled major *CentO* arrays in the pseudomolecules.

We did not detect clear peaks in the other seven centromeres (Figure 1A). The presumed repetitiveness of the sequences in the remaining seven centromeres may prevent detection of the expected sequence read enrichment, since reads of repeated sequences cannot be mapped to specific locations. Four of these seven centromeres (*Cen1*, *Cen2*, *Cen6*, and *Cen11*) had >600-kb *CentO* repeats, and the other three (*Cen3*, *Cen9*, and *Cen12*) had >400-kb *CentO* repeats. In addition, over 70% of the sequence reads that were not mapped to specific locations due to their high copy number or multiple identical matches had significant hits (above the cutoff) in the centromeres.

To estimate the read densities of “unmappable” repetitive subregions for these seven centromeres, we made the simplifying assumption that their densities would be the same as immediately flanking “mappable” subregions. Within each 20-kb window, we identified subregions that are either highly repetitive (≥50 copies) or represent identical duplicates in the genome. We then calculated the size of mappable sequences for each 20-kb window by subtracting the length of the above unmappable subregions from 20 kb. For each window, we extrapolated the read count of the mappable sequence over the whole 20-kb window. As a control, we went through the same procedure for approximately 2 Mb of sequences flanking the crossover-suppressed domain on each chromosome arm. This read-density plotting revealed the boundaries for four additional centromeres, including *Cen1* (∼880 kb), *Cen3* (∼1,210 kb), *Cen9* (∼500 kb), and *Cen12* (∼390 kb) (Figure 1B). However, we were not able to define the boundaries of the CENH3-binding domains for *Cen2* (∼630 kb *CentO*), *Cen6* (∼880 kb *CentO*), and *Cen11* (∼2 Mb *CentO*). Because the CENH3-binding domains in most rice centromeres span 500–1,000 kb and all rice centromeres are known to contain *CentO* [14], we predict that the CENH3-binding domains of *Cen2*, *Cen6*, and *Cen11* contain mainly unmappable *CentO* repeats.

Finally, we used real-time PCR to verify the CENH3 binding at five of the centromeres, including *Cen1*, *Cen3*, *Cen9*, *Cen10*, and *Cen12*. Three to five primer pairs (primer numbers 72–109, Table S1) were designed to target the read-density peaks in the vicinity of each defined border of the CENH3-binding domains (indicated by arrows in Figure 1B). These primer pairs showed 3.7-fold to 57.4-fold enrichment of ChIPed DNA over the mock control (*p* = 0.03 – 7.8 × 10^−7^, one-tailed Student *t*-test; Figure S3). These data supported our defining of the CENH3 boundary by using the normalized ChIP-Seq profiles.

### Ancient Duplication of a Centromere Region

**Figure 2:**
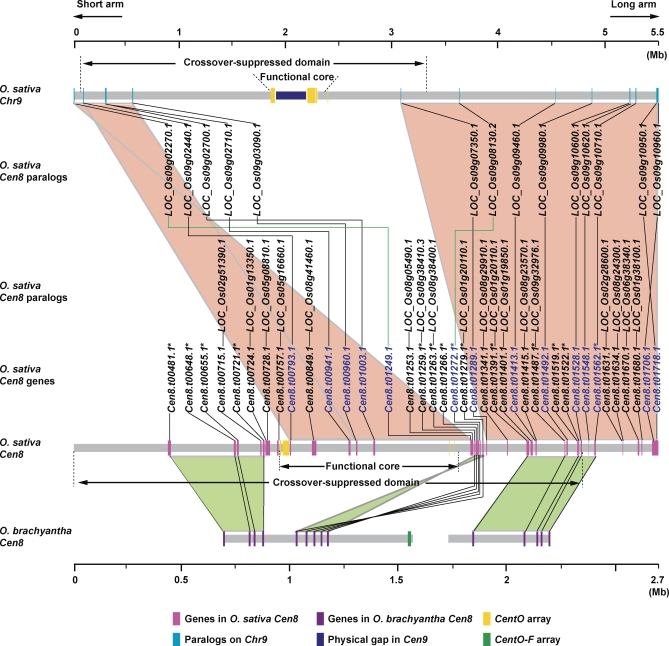
Illustration of Duplication of *Cen8* and *Cen8*-Associated Genes on Chromosome 9 The approximately 2.7-Mb Nipponbare *Cen8* region is displayed at twice the scale as that of the 5.5-Mb chromosome 9 region. A total of 32 active genes located in *Cen8* were used for comparative mapping and analysis. Twenty-seven of the paralogs have cognate ESTs or full-length cDNAs (http://rice.plantbiology.msu.edu/); expression of the others was confirmed by RT-PCR (unpublished data), with the exception of *Loc_Os09g10620.1*, whose transcription was not tested. Two syntenic blocks with five and nine paralogs of *Cen8* genes (gene names in blue), respectively, were identified on chromosome 9, separated by a distance of approximately 2.5 Mb containing the chromosome 9 centromere core. Another 18 *Cen8* genes had paralogs elsewhere in the genome (displayed below the chromosome 9 paralogs). The order of Nipponbare *Cen8* gene orthologs (indicated by an asterisk [*]) flanking the O. brachyantha satellite array is shown at the bottom [24].

A genome-wide duplication event is thought to have occurred during rice genome evolution because extensive regions of syntenic paralogous genes exist between several chromosome pairs [21,22]. If rice centromeres have ancient origins and have stable locations, they may therefore also exist as pairs of paralogs. Synteny of paralogous centromeric regions might be obscured, though, because centromeres are frequent sites for rearrangements [23]. For example, *Cen8* has been subject to a rearrangement and positional shift compared with relatives that diverged about 5 and 10 million years ago (Mya) [24,25] (Figure 2). To investigate the divergence of paralogous centromeric regions, we sought to identify any possible homologous segments of *Cen8* in the rice genome by searching paralogous genes that share significant sequence similarity to the 29 active genes (21 with expressed sequence tag [EST]/cDNA and eight supported by reverse-transcriptase PCR (RT-PCR)) located in the 1-Mb sequence spanning *Cen8* (Table 1). We identified apparently functional paralogs for 17 of the 29 genes (Table S2). Twelve of these 17 *Cen8* genes (*Cen8.t00715.1* and *Cen8.t00724.1* to *Cen8.t01259.1*) showed 49%–97% protein identity with their respective paralogs (Figure 2). These 12 pairs of genes are highly conserved in the number of exons and the size of internal exons. Furthermore, five of these 12 *Cen8* genes, together spanning a region of 827 kb that covers over 94% of the functional core, best matched another set of five genes scattered over a 544-kb region on the short arm of chromosome 9 (*Loc_Os09g02270.1* to *Loc_Os09g03090.1*, Figure 2). The major portion of this segment on chromosome 9 (∼471 kb) is located within the crossover-suppressed domain of *Cen9* and is approximately 1,280 kb away from the *CentO* array of *Cen9*. The structural changes between the two duplicated segments are profound, including rearrangements of the conserved five gene pairs, as well as a lack of conservation for the majority of the active genes: 12 out of the 17 genes from chromosome 9, and 24 out of the 29 genes from *Cen8*.

**Table 1:**
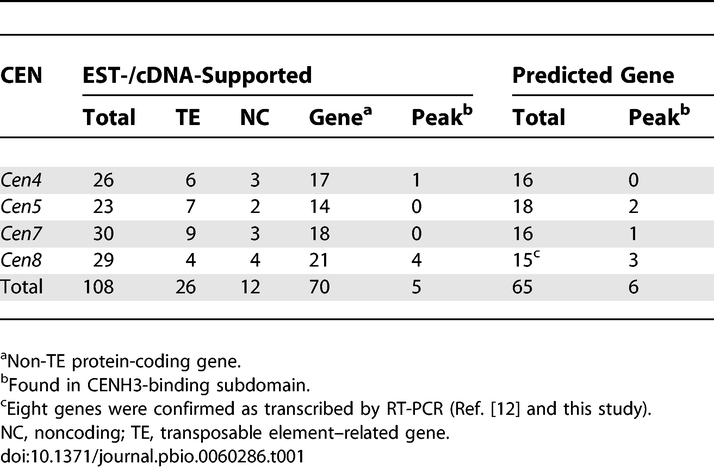
Genes and Noncoding Regions Identified from Four 1-Mb Sequences Spanning Four Rice Centromeres

To define the boundaries of this duplicated region, we searched for nontransposable element (non-TE) genic matches to the rice genome within the 2,481 kb of DNA that flanks the short arm (1,259 kb) and long arm (1,222 kb) sides of the 1-Mb *Cen8*-spanning region [12]. The active genes from the 1,259-kb short-arm side had no matches in the vicinity of the 544-kb syntenic region on chromosome 9. However, nine genes (*Cen8.t01272.1* to *Cen8.t01718.1*) from the 1,222-kb long-arm side had best matched paralogs in a 2,394-kb block on the long arm of chromosome 9, while another 11 *Cen8* genes had best-matched paralogs elsewhere (*Loc_Os08g38400.1* to *Loc_Os01g38100.1*, Figure 2). The two syntenic blocks on chromosome 9 are separated by a region of approximately 2,487 kb (including the 302-kb gap) that contains the functional core of *Cen9*. Thus, *Cen8* structurally resembles a deletion product derived from the *Cen9* region (Figure 2). These syntenic blocks suggest that centromere function has remained within a region of approximately 3 Mb since the divergence of *Cen8* and chromosome 9, although the actual sequences of the functional centromere core have changed dramatically.

Except for paralog *Loc_Os09g10620.1*, which was not assayed by reverse-transcriptase PCR (RT-PCR), all of the other 19 (9 + 11 − 1) paralogs for genes from the long-arm side are active genes with cognate ESTs/cDNAs or positive RT-PCR (primers 22–29 in Table S1). We evaluated the divergence of expression between eight of the 14 syntenic gene pairs by real-time RT-PCR using locus-specific primers (primers 40–71 in Table S1). RT-PCR results confirmed that these genes are transcribed in all five cDNA samples, including leaves, roots, etiolated leaves/shoots, calli, and panicle (unpublished data), suggesting no difference in tissue specificity for these gene pairs. Nevertheless, real-time RT-PCR assays revealed significant alteration in levels of expression between these gene pairs in one to five cDNA samples (Figure S4). Genes with relatively higher or lower levels of expression are not biased towards the *Cen8* or chromosome 9 segment. For example, gene *Cen8.t00793.1* had a significantly higher level of expression than its paralog *Loc_Os09g02440.1* in three of the tissues; in contrast, gene *Cen8.t00960.1* had a markedly reduced level of expression compared with *Loc_Os09g03090.1* across all the five tissues.

To determine whether this duplication is confined to Oryza sativa (2*n* = 24, AA genome) or shared by other *Oryza* species, we compared protein sequences from these 14 pairs of genes to approximately 146 Mb of genome survey sequences in GenBank representing 11%–13% genome coverage from two non-AA species: O. brachyantha (2*n* = 24, FF genome) and O. granulata (2*n* = 24, GG genome). For the *Cen8* genes, we have identified five putative orthologs in O. brachyantha and three in O. granulata; for the chromosome 9 genes, we have identified five putative orthologs in O. brachyantha and four in O. granulata (Table S3). Therefore, this duplication predated the approximately 10 Mya of divergence between O. sativa and the most distantly related *Oryza* species O. granulata [25]. We used the YN00 program available in PAML 4 [26] to obtain an approximate estimation of age for the divergence of *Cen8* genes and their chromosome 9 paralogs, using a substitution rate of 6.5 × 10^−9^ per synonymous site per year in rice [27]. These 14 pairs of duplicated genes had an average *K*s of 0.7717 ± 0.1927 (mean ± standard deviation [SD]), corresponding to a divergence time of 59.4 (±14.8) Mya (Table 2). This date is similar to the estimated age of 53–76 Mya for the divergence time of the duplicate chromosomes in a whole-genome duplication event that is hypothesized to have occurred prior to the radiation of grasses [22]. Synteny of this age between chromosome 8 and chromosome 9 was previously described as part of this whole-genome duplication, but these reports did not include the centromere region [21,22].

**Table 2:**
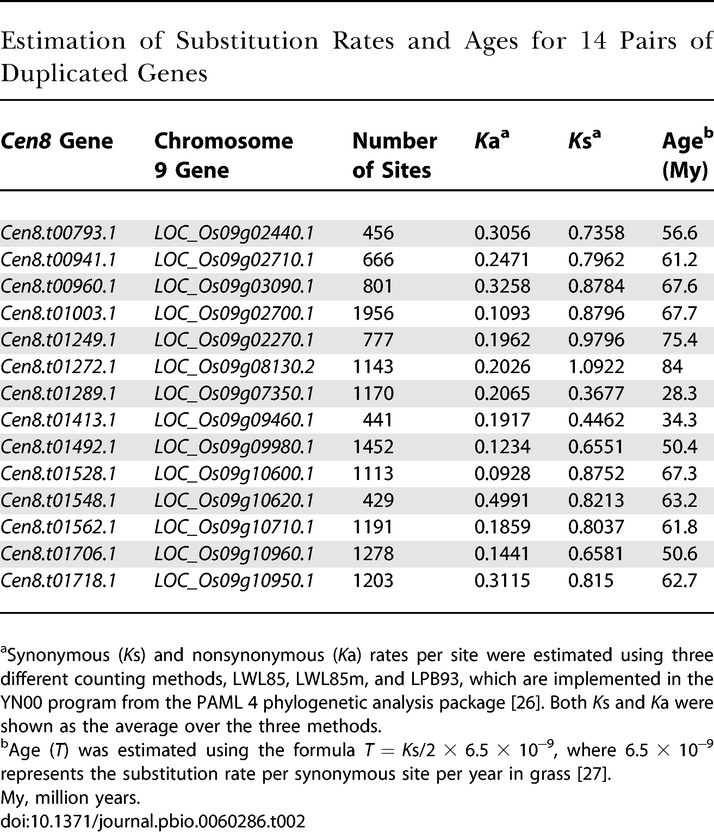
Estimation of Substitution Rates and Ages for 14 Pairs of Duplicated Genes

### CENH3 Occupies Distinct Subdomains in Rice Centromeres

**Figure 3:**
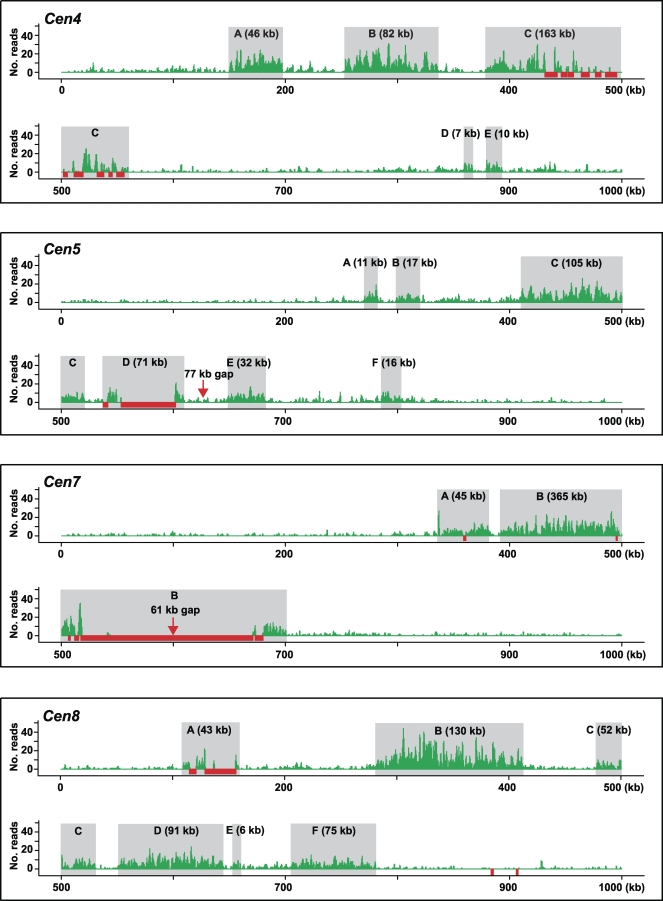
Profiles of CENH3 Binding in Four Rice Centromeres The number of ChIP sequence reads is plotted along its position in the selected 1-Mb region spanning the functional core for each of the four centromeres, using 1-kb sliding windows every 500 bp. ChIP-Seq peaks were defined as those that have five or more reads in a 1-kb window, corresponding to a *p*-value of 1 × 10^−4^ or 2 × 10^−4^, depending on chromosome. Red bars indicate *CentO* satellite arrays. Subdomains showing CENH3 binding peaks of >5 kb are highlighted in grey.

Our previous real-time PCR- and hybridization-based assays revealed that the occupancy of CENH3 in the functional domain of rice *Cen8* [11] and *Cen3* [13] is not continuous. Nevertheless, the extent of CENH3 binding and its positioning relative to that of the canonical histone H3 remained elusive. In the present study, we observed marked enrichment of ChIP sequence reads within *Cen4*, *Cen5*, *Cen7*, and *Cen8*, thus enabling us to profile the CENH3 binding at these four centromeres at a much higher resolution using 1-kb sliding windows (Figure 3). This plotting revealed two to six major subdomains of CENH3 binding within the four centromeres, ranging in size from a 6-kb subdomain in *Cen8* up to a subdomain of approximately 365 kb in *Cen7*. *Cen4*, *Cen5*, and *Cen8* shared a similar pattern of CENH3 occupancy: each had five to six major CENH3 subdomains that together occupied 38%–49% of the entire functional core. In contrast, *Cen7* had a distinctive CENH3 organization in which its 420-kb functional core was predominantly occupied by the two major CENH3 subdomains of 45 kb and approximately 365 kb, although we can not determine whether CENH3 binding is continuous over the central *CentO* array. We were unable to identify any sequence motifs that appeared to be putative boundary elements or that otherwise could predict the boundaries of these subdomains.

### CENH3 Subdomains Are Depleted of Genes

Our previous analyses showed that active genes occur in the functional domains of *Cen3* and *Cen8*, but that gene density is reduced relative to flanking pericentric regions [12,13]. Low gene density might reflect a preferential association of CENH3 nucleosomes with nongenic DNA sequence, or it might simply reflect the random accumulation of transposons and satellites in a crossover-free region; in which case, we would expect CENH3 subdomains to include genes in proportion to the fraction of the total functional domain that these subdomains occupy. However, our earlier data provided only limited information about the distribution of CENH3 nucleosomes relative to genes.

**Figure 4:**
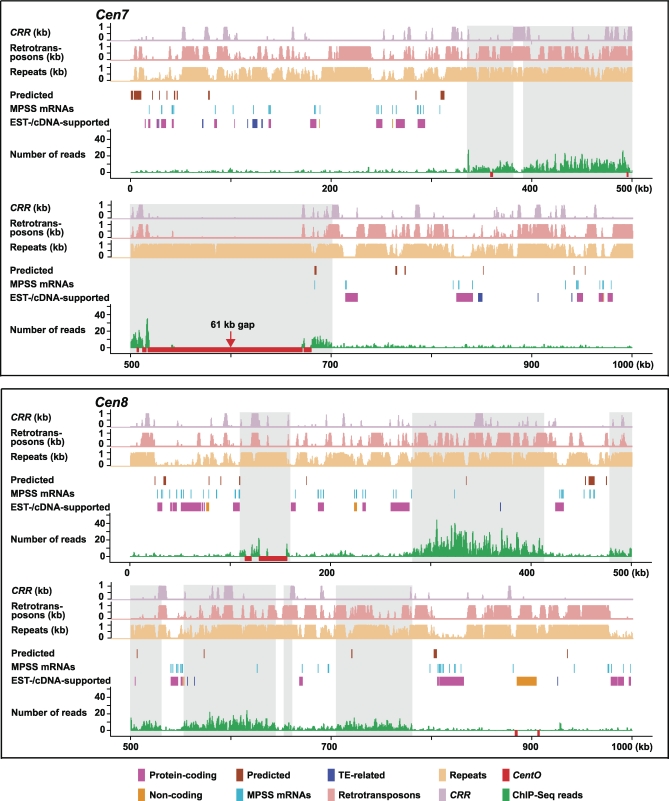
Distribution of Genes, Repeats, and MPSS mRNA Signatures in *Cen7* and *Cen8* Repeats are defined as those that have 20 or more copies in the genome and plotted using a 1-kb window. CRR centromere-specific retrotransposons and other retrotransposons are plotted separately from the remaining repeats. Transcribed regions supported by ESTs or cDNAs are annotated as non–TE-related protein-coding genes, TE-related genes, or noncoding regions. Predicted genes (http://rice.plantbiology.msu.edu/) and MPSS mRNA signatures (http://mpss.udel.edu/rice/) were downloaded from public databases. Major CENH3-binding subdomains (>5 kb in size) are highlighted as in Figure 3. Two MPSS tags within the highlighted subdomains of *Cen8* fall within individual 1-kb windows that are not enriched in CENH3, whereas the single MPSS tag in *Cen7* is in a CENH3-enriched window.

We previously identified 16 active genes from the functional core of *Cen8* that were enriched for H3K4me2 [12]. CENH3 is sufficiently dissimilar from H3 so that the H3K4me2 antibody should only recognize H3, but not CENH3, thus linking these active genes to regions of H3 nucleosomes. To verify this inference, we conducted a comprehensive annotation for transcribed regions from the set of four 1-Mb sequences that span the functional core of *Cen4*, *Cen5*, *Cen7*, and *Cen8*. By using over 1.2 million publicly available rice ESTs and full-length cDNAs (fl-cDNAs), we identified a total of 108 regions as being transcribed, including 70 regions harboring non–TE-related protein-coding genes (Tables 1 and S4). Strikingly, only five of these regions were located in the CENH3 subdomains (Figures 4 and S5; Table 1), including three TE-related genes in *Cen8* (*Cen8.t13171.1*, *Cen8.t13349.1*, and *Cen8.t13363.1*) with unspliced ESTs. Real-time PCR analysis confirmed that these five genes indeed resided within the CENH3 subdomains (primers 1, 3–4, and 6–12 in Table S1; Figure S6). Excluding gene *Cen8.t01075.1* [12], which overlapped a CENH3 peak in its intron, we used RT-PCR to verify the expression of the remaining four genes across a panel of four different tissues (primers 1–2, 4–5, and 7–10 in Table S1). Three of the four genes were confirmed to be transcribed in at least three different cDNA samples (Figure S7), suggesting that the CENH3-containing nucleosomes are likely to be compatible with transcription. We further mapped a total of 222 massively parallel signature sequencing (MPSS) mRNA tags (17 or 20 bp) to the four centromeres. Most of the tags were mapped to regions corresponding to annotated active genes in the CENH3-lacking subdomains. Only two tags were derived from regions with CENH3 binding, one in *Cen4* (Figure S5) and the second one in *Cen7* (Figure 4).

Likewise, hypothetical genes solely predicted by gene finders were also significantly biased away from the CENH3-binding subdomains. Out of the 65 genes predicted from these 4-Mb sequences, only six were found to reside within the CENH3 subdomains (Table 1). Primers were designed for three of these hypothetical genes (primers 15–19 and 21 in Table S1), and real-time PCR analysis verified their association with CENH3 binding (Figure S6). Of the six genes, *Cen8.t00761.1* was previously known to be inactive in all of the four tissues [12]; we performed RT-PCR on three other genes (primers 13–14 and 17–20 in Table S1), confirming *Loc_Os08g22389.1* as the only active gene (Figure S7).

A close examination of these active genes (supported by ESTs and cDNAs) within the four 1-Mb centromeric regions revealed two distinct patterns of organization. *Cen4*, *Cen5*, and *Cen8* each had six to 13 active genes within the core, and 11 out of the 14 intervening regions between major CENH3 subdomains each had at least one active gene. However, we did not identify any active gene in the approximately 420-kb core of *Cen7*, which showed a much higher proportion of CENH3 occupancy than the other three centromeres. The 18 active genes from the 1-Mb region spanning this centromere were all present in the flanking regions.

In addition, of the 287 MPSS small RNA signatures mapped to the four 1-Mb sequences, over 96% matched sequences from the CENH3-lacking subdomains, reflecting a pattern highly consistent with what we have seen for the centromere-derived mRNA and cDNA sequences. Collectively, these data show that genes are almost entirely confined to the CENH3-lacking subdomains in rice centromeres (Figures 4 and S5).

### Nonfunctional Genes in the CENH3 Subdomains

A centromere might evolve from an ordinary genomic region via a neocentromeric intermediate [28]. The lack of genes and transcription in the CENH3 subdomains is consistent with two alternative hypotheses regarding the origin and evolution of rice centromeres. The CENH3 subdomains originally might have contained a similar density of active genes as the flanking CENH3-lacking subdomains, but most of these genes have lost their function and degenerated during centromere evolution. According to this hypothesis, for the genes that were functional prior to the centromere formation, we would be able to identify a nonfunctional gene copy or gene fragment in the CENH3 subdomains of the present-day centromeres. Alternatively, the original CENH3 subdomains might have been quite small and thus contained few active genes. During centromere evolution, individual CENH3 subdomains might have expanded as a result of TE insertion, satellite amplification, or both.

To distinguish these possibilities, we looked for instances where active genes from outside of centromeres have homologs in the centromeric regions, which we examined for evidence of deterioration. We first divided the 372-Mb finished sequence of the rice genome into two portions: the 4-Mb sequences corresponding to the four centromeres as discussed above and the remaining 368-Mb DNA. We then compared all the non–TE-related active genes (with cognate ESTs or cDNAs) from the 368-Mb DNA to the 4-Mb centromere sequences by Tblastn. We identified eight regions in the CENH3 subdomains that showed sequence similarity to active genes located elsewhere. We manually checked the sequence alignments between these eight centromere regions and their respective homologous genes outside of the centromeres, and found that the centromeric homologs were associated with a premature stop codon (three genes), frame shift (one gene), or truncation of coding sequence (three genes). The presence of a premature stop codon or frame shift is strong evidence for a pseudogene status; for the genes whose parental genes have only a single coding exon, truncation of the CDS also suggests a pseudogene status [29]. Based on this analysis, seven genes can be classified as pseudogenes (Table S5).

Finally, for five of the eight centromeric genes, including *Cen7.t12284.1*ψ for which no sign of deterioration was detected, we designed primers (primers 30–39 in Table S1) from the putative exons for RT-PCR analysis. None of the primer pairs yielded amplification in any of the four tissues (unpublished data). Therefore, we conclude that all these eight genes from the CENH3 subdomains are pseudogenes. For comparison, we similarly searched for sequence similarity between active genes located outside of the four centromeres and the CENH3-lacking subdomains of *Cen8*, and found 40 such regions (43 *Cen8* regions – 3 in CENH3-binding subdomains; Table S2). Twenty-three of them matched: intergenic regions (15 − 3), introns of active genes (2), inactive genes (6), or noncoding regions (3), and all but two of them displayed features of deterioration, resembling those from the CENH3 subdomains. The fact that CENH3 subdomains have far fewer active genes, predicted genes, and pseudogenes than CENH3-lacking subdomains suggests that the regions to which the CENH3 was deposited during centromere formation originally had few genes.

## Discussion

Our genome-wide determination of CENH3 binding has mapped the boundaries of nine of the 12 rice centromeres despite their location predominantly in highly repetitive *CentO* satellite sequences. Within four of these centromeres, we mapped discontinuous CENH3-binding subdomains ranging in size from 6 kb to approximately 365 kb. Previously, mapping of discontinuous CENH3-binding at the sequence level has only been possible in two human neocentromeres [8,9] because of their lack of highly repetitive sequences. The CENH3 subdomains mapped here from four natural centromeres resemble those from neocentromeres in that they vary in size and spacing. Remarkably, these regions are deficient in both genes and pseudogenes compared with adjacent CENH3-lacking subdomains, strongly suggesting that they were gene poor at the time CENH3 became established in these subdomains. DNA sequences in rice such as retrotransposons are typically removed in a few million years through unequal homologous recombination and illegitimate recombination [30,31]. We therefore cannot formally exclude the possibility that genes and pseudogenes have been preferentially removed from CENH3-binding subdomains, but we know of no reason to expect that these processes would selectively remove pseudogenes from the CENH3-binding subdomains but not from the interspersed CENH3-lacking subdomains of *Cen8*. We therefore think it is more likely that CENH3-binding subdomains are preferentially established in regions depleted of genes.

New centromeres have been proposed to arise from neocentromeres that subsequently accumulate satellite sequences [28]. *Cen8* is similar to neocentromeres in having a substantial amount of unique sequences, with only approximately 70 kb of the rice centromeric satellite *CentO* in a 750-kb CENH3-binding region that includes active genes [11,12]. *Cen8* thus resembles a transition stage between a neocentromere and a centromere composed of satellite repeats [11]. Recently, this view has been challenged based on evidence that the orthologous regions to *Cen8* in the related wild rice species O. brachyantha and O. officinalis have an inversion of marker order [24]. These authors suggested that *Cen8* was instead derived by recent rearrangement from an ancestral centromere with a typical large satellite array. An ancient satellite array can also be inferred from comparison with the paralogous region that includes *Cen9* (Figure 2) and with *Cen8* from the closely related rice species O. punctata, in which CENH3 binds to a *CentO* array estimated to be 1.4 Mb [32]. These observations suggest that a conventional satellite array served as the centromere on chromosome 8 from prior to the divergence of grasses 50–70 Mya until after the divergence of closely related rice species approximately 5 Mya. However, the existence of an ancient satellite array that was subsequently lost can be viewed as supporting a neocentromere-like origin of the present rice *Cen8*, which occupies largely nonsatellite sequences that presumably did not function as a centromere prior to rearrangement sometime within the last approximately 5 Mya. *Cen8* differs from mammalian neocentromeres, however, in being located within a few megabases of the ancestral satellite array and perhaps retaining a small piece of that array, whereas mammalian neocentromeres form with no satellite sequences at many locations that are cytologically distant from the previous centromere. Despite these differences, the similarities between *Cen8* and neocentromeres suggest that common processes underlie the formation of both.

Like *Cen8*, neocentromeres have appeared in gene-poor regions following chromosome rearrangements that delete or disrupt native centromeres. For example, rare human centromere “shifts” are associated with simultaneous deletion of centromeric alpha-satellite [4]. Two neocentromeres in 15q24–26 were found near rearrangement breakpoints [33], suggesting a possible direct role for rearrangements in both neocentromere and *Cen8* formation, perhaps through chromatin remodeling associated with DNA repair. In maize, an inversion with one break in centromeric satellite generated a new centromere adjacent to but not including one portion of the split satellite array [34]. Other neocentromeres have been found in large (0.8–3.9 Mb) gene-free or gene-poor regions [35,36], and the discontinuous binding of CENP-A in one neocentromere was found in intergenic subregions of an otherwise relatively gene-rich region [9]. These observations echo the gene depletion we see in the CENH3-binding subdomains of four native rice centromeres, and strongly suggest that gene activity, although not completely incompatible with centromere function, is detrimental to neocentromere establishment and centromere maintenance. This is consistent with the facts that regional centromeres in plants and animals typically occupy nongenic satellites, and that the short centromeres of several unicellular eukaryotes also reside in smaller (4–18 kb) gene-free regions [37–39]. Gene transcription results in nucleosome replacement [40], and transcription disrupts the single centromeric nucleosome of a budding yeast “point” centromere [41]. In *Drosophila*, CENH3 nucleosomes have been shown to be heterotypic tetramers that wrap only a single turn of DNA, in contrast to octameric H3 nucleosomes [42], and this may make them more susceptible to displacement by transcription. CENH3 nucleosomes can be normally incorporated into euchromatic regions [19], and transcription may be important for evicting them so that they can be degraded by proteasomes [43]. Many satellites are also transcribed, but most such transcripts are processed into short interfering RNAs (siRNAs) that result in H3 methylation and silencing, keeping satellite transcription at very low levels [32, 44–46]. Regions of low gene activity may therefore favor retention of enough CENH3 nucleosomes to establish and maintain kinetochore function.

Neocentromeres are smaller and have less CENP-A than normal human centromeres [47], suggesting that they must expand if they are to become successful mature centromeres. The *Cen8* region has expanded by segmental duplication [48] and by insertion of retrotransposons, which comprise nearly a third of *Cen8* [12] and which are enriched relative to the orthologous region of O. brachyantha [24]. New retrotransposons are likely to become silenced and methylated on histone H3K9, which is incompatible with kinetochore function [10]. However, once retrotransposons mutate to nonfunctionality, silencing may be lost and degenerating retrotransposons may contribute to the growth of nongenic CENH3-binding regions, and hence to expanded centromere variants that become rapidly fixed through centromere competition in female meiosis [1].

Regional centromeres that consist of unique sequences like neocentromeres and *Cen8* appear to be short-lived, since most regional centromeres are composed of satellite repeats. Eventual acquisition of the 150–180-bp satellites that underlie both CENH3 and H3 nucleosomes in most regional centromeres may be favored because of their ability to order nucleosomes into regular arrays [49]. The occurrence of 180-bp satellites in classical maize neocentromeres (knobs), which lack CENH3 [50], strongly suggests that satellites are favored for their ability to organize H3 octameric nucleosomes. A densely packed rigid structure of H3 nucleosomes imposed by satellites [51] may help to orient and distribute the anaphase forces of the 10–100 microtubules that are bound to centromeric chromatin in a typical regional centromere [52]. Since satellite arrays also have low transcriptional activity and are readily expandable, they may provide the same advantages for centromere function as gene-depleted unique sequences while also ordering nucleosomes. The exact mechanisms by which satellite arrays can expand and become homogenized are not well understood, but they probably include unequal crossover and gene conversion events that yield new centromere variants at an accelerated frequency. Competition between these variants in female meiosis [1] may underlie rapid replacement of one type of satellite with another, as has been observed between wild rice species diverged from O. sativa by approximately 5 and 10 Mya [53]. Satellites acquired by neocentromeres or other unique-sequence centromeres like *Cen8* may eventually come to dominate these centromeres, thereby converting them into mature regional centromeres.

## Materials and Methods

### ChIP and 454 sequencing.

ChIP was performed as previously described [11]. Approximately 50 g of rice leaf tissue were used in ChIP to obtain approximately 3 μg of immunoprecipitated DNA for sequencing. The ChIP DNA was subjected to modified sequencing pipeline on Roche's GS 20 sequencing platform [54] after the concentration and the size distribution of the ChIP DNA were accurately measured by Agilent's Bioanalyzer and Invitrogen's Qubit fluorometer. DNA fragmentation step was omitted for Roche's library construction based on the size of the ChIP DNA. DNA sample was end-repaired and phosphorylated before being ligated to Roche's genomic sequencing adaptors. Single-stranded library fragments were collected by following the manufacturer's instruction. The concentration and the size distribution of the single-stranded library was measured by Agilent's Bioanalyzer and appropriate amount of the library was mixed and hybridized with Roche's DNA capture beads. DNA bound beads were amplified and enriched and then were loaded onto GS 20 platform according to modified manufacturer's instruction.

### Mapping of 454 sequence reads.

We generated a total of 325,298 high quality reads. The majority of the sequence reads (94%) had a length between 80 and 130 bp. We first used Blastn to find *CentO*-containing sequences from all ChIP 454 sequence reads. The rice variety Nipponbare pseudomolecules (http://rice.plantbiology.msu.edu/, version 5) contained 6,003 hits that aligned with *CentO* consensus over at least 20 bp, only 24 of which were present outside of the genetically defined centromeres. Therefore, we defined the *CentO*-containing reads as those that had a minimum of 20-bp alignment with *CentO*. We then used Megablast to map the remaining *CentO*-less reads to the version 5 pseudomolecules, at the cutoff of ≥97% identity and ≥90% coverage of the read length. We found that 4.3% of the reads had no hits or had hits below the cutoff, and were excluded from further analysis. Of the remainder, we did not assign map locations for 46,436 reads that either had two or more hits with the same identity and coverage, or had 50 or more copies but lacked a perfect match. By retaining only the best hit for reads with fewer than 50 copies and the perfect match for those with a higher copy number, we were able to assign map locations for 151,810 of the reads, accounting for 46.7% of the total dataset (Figure S2).

### Data plot, normalization, and peak detection.

To plot the distribution of 454 reads, we split each chromosome into 20-kb windows, spaced every 10 kb, and plotted the number of reads mapped to each window along its chromosome coordinate. Centromeres have a much denser distribution of repetitive DNA than chromosome arms, so reads derived from centromeric regions are less likely to be mapped to distinct locations. To address this mapping bias, we screened for less repetitive, mappable sequences for each window and used the read count over these mappable sequences to calculate a read density representing the relative enrichment.

For each chromosome, we extracted a portion of the *CentO*-masked pseudomolecule that covers the entire genetically defined centromere and extends approximately 2 Mb into the flanking region on each side. The extracted sequences were split into 60-bp bins, spaced every 30 bp, which were blasted against the whole rice genome. For each bin, we retained hits with alignment of ≥30 bp and calculated its copy number. Overlapping bins with less than 50 copies were concatenated. We further tried to identify regions that have at least one duplicate of 100% identity elsewhere in the genome. We parsed out bins that had alignments of ≥30 bp of 100% identity to more than one location, and overlapping bins were concatenated. We aligned them back to the genome to identify those that have at least one perfect duplicate match of ≥130 bp, since over 98% of the reads are <130 bp in length. Regions that had fewer than 50 genomic hits but lacked a perfect duplicate were merged with the locations anchored by the mapped ChIP-Seq to form the mappable regions.

We estimated the read density, which represents the number of reads in a 20-kb mappable region, using the above 20-kb windows according to the following formula:

Read density = ([Number of reads − 10]/mappable sequences in kilobases) × 20 kb, where 10 is the 75th percentile for the number of reads in the 20-kb windows from both chromosome arms, which are the same for all chromosomes.

We also plotted the read count using 1-kb window size for chromosome 4, chromosome 5, chromosome 7, and chromosome 8. To identify a subset of 1-kb windows that are enriched for ChIP-Seq reads, for each chromosome, we generated expectation distributions of read counts under the null hypothesis of no enrichment. This was accomplished by randomly assigning each mapped ChIP-Seq read to a unique chromosome position ten times and calculating the nominal *p*-values for each 1-kb window. Enriched 1-kb windows were defined as those that had a *p*-value of ≤1 × 10^−4^ (chromosome 5 and chromosome 7) or 2 × 10^−4^ (chromosome 4 and chromosome 8), corresponding to five read counts.

### Annotation of genes and transcribed regions.

We used a total of 32,129 full-length cDNAs (http://cdna01.dna.affrc.go.jp/cDNA) and over 1.2 million ESTs (GenBank dbEST) from rice to identify regions of transcription from four 1-Mb centromeric regions according to the published procedure [12]. These four 1-Mb regions span the cores of *Cen4*, *Cen5*, *Cen7*, and *Cen8* as revealed by the current study. Transcribed regions were further broken down into non–TE-related protein-coding genes, TE-related genes, and noncoding regions. Additional regions of transcription were identified by using rice MPSS mRNA and small RNA signatures [55] as described [13], where only signatures with a single perfect match in the genome (to a given centromere) were retained. Finally, predicted genes without EST/cDNA evidence for transcription were downloaded from the rice genome annotation project database (http://rice.plantbiology.msu.edu), and the expression of those present in *Cen8* was tested by RT-PCR, either previously [12] or as part of this study.

### Identification of centromeric regions that show homology to active genes elsewhere.

We used Tblastn to compare the above four 1-Mb centromere sequences to a total of 32,816 putative non–TE-related protein sequences representing 23,570 active genes from the rest of the genome. By this comparison, we tried to identify whether any of the active genes in the centromeres has a functional copy elsewhere in the genome. We also wanted to understand whether there are major differences between the CENH3 subdomains and the CENH3-lacking subdomains regarding the distribution of decayed genes or gene fragments (pseudogene) whose parent genes are outside of centromeres. For a match to be considered significant, we required an identity of ≥35%, a coverage of ≥50% of the parent gene, and an alignment length of 50 or more amino acids (aa), or a coverage of <50% but an alignment length of ≥100 aa.

The alignment output between the genomic sequences from the centromeres and protein sequences from active genes elsewhere were manually checked for instances of gene deterioration. By comparison to the parent gene, the homologous region from the centromere was classified as representing a pseudogene if any of the following features of deterioration were detected: a premature stop codon, a frame shift caused by insertion or deletion, or a 3′ or 5′ truncation of the coding sequence. In addition, regions confirmed to be not expressed by RT-PCR were also annotated as pseudogenes. If the homologous region from the centromere was already annotated as a gene, a further comparison was made to reveal any changes in gene structure.

### RT-PCR, real-time PCR, and real-time RT-PCR.

We used RT-PCR to test the expression of genes present in the CENH3 ChIP-Seq peaks, including four genes with unspliced ESTs (primers 1–2, 4–5, and 7–10), three hypothetical genes (primers 13–14 and 17–20), and five pseudogenes (primers 30–39), as well as another four hypothetical genes (primers 22–29) that best matched *Cen8* active genes. Primers were designed to have a length between 21 and 29 bp, with an annealing temperature from 60.1 to 66.7 °C (Table S1). We isolated total RNA from four different rice tissues or treatments, including leaves, roots, etiolated leaves/shoots, and calli, and performed RT-PCR as described [12].

We designed ChIP-PCR primers to verify the CENH3 binding in 19 predicted ChIP-Seq peaks from five centromeres (Figure S3) and eight genes also in the ChIP-Seq peaks (Figure S6). Real-time PCR analysis was used to determine the relative enrichment of CENH3-associated sequences in the bound fraction over the mock control. PCR reactions were carried out in triplicates using the DyNAmo HS SYBR Green qPCR kit (MJ Research) and run at 95 °C for 15 min, followed by 45 cycles of 95 °C for 10 s, 62–65 °C for 30 s, and 72 °C for 30 s. We used three active genes as negative controls that yielded similar results, including *Cen8.t00421.1* and *Cen8.t00479.1* [12], as well as *LOC_Os01g57730.1*. For each primer pair, we calculated the relative fold enrichment (RFE) as described [12], using *Cen8.t00421.1* as the reference gene.

We used real-time RT-PCR to compare the transcription level between eight pairs of duplicated genes from *Cen8* and chromosome 9. For each gene pair, locus-specific primers were designed based on the alignment between the coding sequences from both copies, ensuring that each *Cen8*-locus primer must have at least five base pairs difference from the corresponding chromosome 9-locus primer, both with similar annealing temperatures (Table S1). Real-time RT-PCR was conducted following the above procedure, where the previously described four cDNA samples were tested, plus an additional cDNA sample from 3-d-old panicles. We used real-time PCR on genomic DNA to evaluate whether there is a difference in the amplification efficiency between the primer pair targeting the *Cen8* copy and that targeting the chromosome 9 copy. For each gene pair, we obtained the PCR cycle threshold (CT) difference from the genomic DNA template, expressed as ΔCT-gDNA = CT (*Cen8* copy) − CT (chromosome 9 copy), and used this difference to normalize individual ΔCT-cDNA (CT difference from each cDNA sample). A relative fold change of a *Cen8* copy over a chromosome 9 copy in expression was calculated as 2^–ΔΔCT^, where ΔΔCT = ΔCT-cDNA − ΔCT-gDNA. We performed a two-tailed Student *t*-test of CT values at the significant level of α = 0.05.

### Evolutionary analysis of duplicated genes.

For each pair of duplicated genes between *Cen8* and chromosome 9, protein sequences were aligned using the National Center for Biotechnology Information bl2seq, and the resulting alignment was manually checked. The corresponding coding sequences were then aligned using ClustalW (http://www.ebi.ac.uk/Tools/clustalw2/index.html), with the protein alignment as the guide. We used the YN00 program from the PAML package [26] to estimate the synonymous (*K*s) and nonsynonymous (*K*a) substitution rates. We averaged the *K*s and *K*a values from three different counting methods, including LPB93, LWL85, and LWL85m that all account for transition–transversion rate difference [26]. This mean *K*s was used to estimate the divergence time between each pair of duplicated genes, using the formula *T* = *K*s/2 × 6.5 × 10^−9^, where 6.5 × 10^−9^ represents the substitution rate per synonymous site per year in grass [27]. Finally, the divergence time between the *Cen8* and chromosome 9 syntenic block was estimated using the average *K*s from all 14 pairs of genes.

## Supporting Information

Figure S1 Genetic and Physical Maps of Rice Centromeres *Cen3* and *Cen8* were described previously [12,13]. For each centromere, its position on the rice genetic map was defined by a set of cosegregated restriction fragment length polymorphism (RFLP) markers (http://rgp.dna.affrc.go.jp/E/Publicdata.html), which are labeled at the bottom of each gray bar. Red bars indicate *CentO* satellite arrays, shown in greater detail above each chromosome map. Between four and 22 cosegregated RFLP markers were anchored to each centromere. Arrows represent the locations of physical gaps on the current sequence maps. The size for four of the gaps was estimated by FISH, including the *Cen4* gap [15], the first gap in *Cen10* [16], the first gap in *Cen7*, and the second gap in *Cen11* [17]; the remaining gaps were sized by optical mapping [18].
(285 KB PDF)

Figure S2 Mapping of ChIP-Seq Sequence Reads Reads showing alignments of ≥20 bp with *CentO* consensus were classified as *CentO*-containing reads. Megablast was used to map the *CentO*-less reads to the genome. For reads with fewer than 50 copies, the cutoff for assigning distinct mapping locations was a minimal similarity of 97% and a minimal coverage of 90%; but for those with 50 or more copies, only reads with a single perfect match were mapped.
(189 KB PDF)

Figure S3 Verification of the CENH3 Binding Peaks Using Quantitative PCR Assays The relative positions of primers on individual chromosomes are not shown to scale. Blue bars represent physical gaps on the current sequence maps. Primer numbers are shown above each horizontal gray bar and are detailed in Table S1. *Cen8.t00421.1*, an active gene located approximately 605 kb away from the left boundary of the CENH3 binding domain in *Cen8*, was used as the negative control, whose relative fold enrichment was set at 1 and used as the baseline (dashed line). For each primer pair, significance of enrichment from antibody-binding fraction over mock treatment was tested using a one-tailed Student *t*-test (α = 0.01, *n* = 3); the corresponding *p*-values are shown above the primer numbers. The relative enrichment is shown as mean ± SD.
(239 KB PDF)

Figure S4 Comparison of the Transcription Levels of *Cen8* Genes and Their Paralogs on Chromosome 9 For each gene pair, relative fold change of the *Cen8* copy over the chromosome 9 copy was calculated in each cDNA sample, normalized by using the real-time PCR cycle threshold (CT) difference on genomic DNA (see Materials and Methods for details). The baseline (relative fold change = 1) stands for the same level of expression between the two copies of a given gene pair. Significance of up- or down-regulated expression was inferred by a two-tailed Student *t*-test (α = 0.05, *n* = 2). The *p*-values of <0.05 are shown above each bar.
(214 KB PDF)

Figure S5 Distribution of Genes, Repeats, and MPSS mRNA Signatures in *Cen4* and *Cen5*
Annotation is as described in legend to Figure 4. Two of the three ESTs that appear in gray-shaded areas correspond to 1-kb windows that are not enriched for CENH3, but that are surrounded by enriched windows. The third EST (corresponding to *Cen4.t09693.1*, positioned at ∼400 kb) is in a CENH3-enriched window (see Figure S6).
(560 KB PDF)

Figure S6 Verification of the CENH3 Binding for Eight Genes Located within the CENH3 Subdomains The five genes on the left side are supported by ESTs, and the other three are hypothetical genes. Relative enrichment was calculated and tested for significance as in Figure S3. Real-time PCR confirmed the CENH3 binding for all eight genes.
(190 KB PDF)

Figure S7 RT-PCR Verification of Transcription of Four *Cen8* Gene Models *Loc_Os08g22389.1* is a hypothetical gene, whereas the other three are TE-related genes supported by unspliced ESTs.
C, cDNA from calli; E, cDNA from etiolated leaves/shoots; G, genomic DNA; L, cDNA from leaves; M, molecular marker; N1, negative control (without adding reverse transcriptase) for leaves and roots; N2, negative control for etiolated leaves/shoots and calli; R, cDNA from roots.
(285 KB PDF)

Table S1 Primers Used in the Study (88 KB PDF)

Table S2 Forty-Three Regions from *Cen8* That Matched Active Genes Elsewhere in the Genome (41 KB PDF)

Table S3 Fourteen Pairs of Duplicated Genes in Nipponbare and Their Respective Orthologs in Two Wild Rice Species (66 KB PDF)

Table S4 Active Genes Identified from Three 1-Mb Sequences Spanning the CENH3 Binding Domain of *Cen4*, *Cen5*, and *Cen7*
(72 KB PDF)

Table S5 Eight Pseudogenes Identified from the CENH3 ChIP-Seq Peaks (59 KB PDF)

## Abbreviations

CENH3: centromeric histone H3 variant
ChIP: chromatin immunoprecipitation
ChIP-Seq: sequencing of chromatin-immunoprecipitated DNA
EST: expressed sequence tag
FISH: fluorescent in situ hybridization
MPSS: massively parallel signature sequencing
Mya: million years ago
RT-PCR: reverse-transcriptase PCR
TE: transposable element
