# Supplementary figures and images for: Intergenic Locations of Rice Centromeric Chromatin
Source: PLoS Biol. 2008 Nov 25;6(11):e286. doi: 10.1371/journal.pbio.0060286 (PMC2586382; doi:10.1371/journal.pbio.0060286)

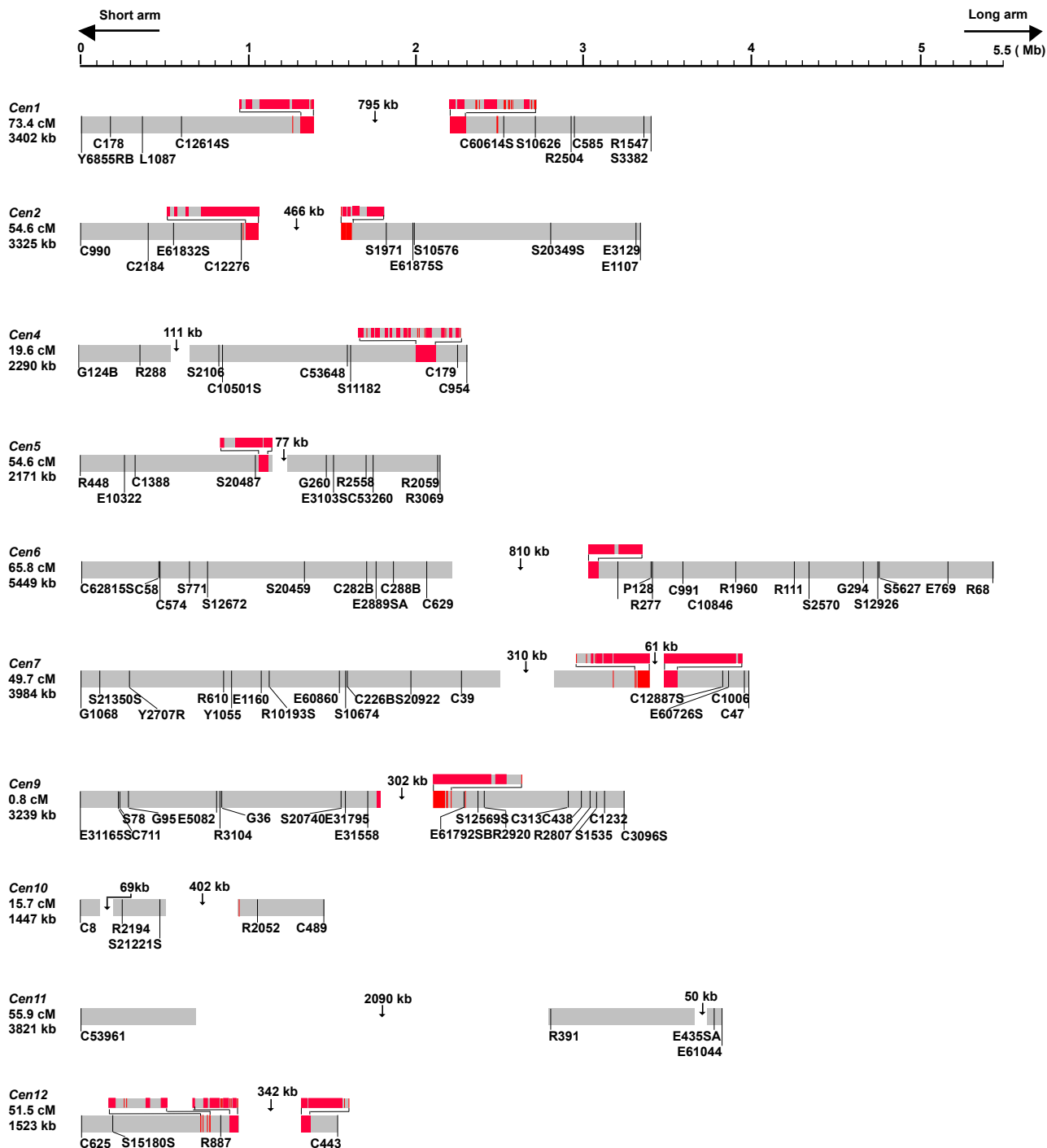

**Figure S1**

Supplement: Figure S1 — Cen3 and Cen8 were described previously [12,13]. For each centromere, its position on the rice genetic map was defined by a set of cosegregated restriction fragment length polymorphism (RFLP) markers (http://rgp.dna.affrc.go.jp/E/Publicdata.html), which are labeled at the bottom of each gray bar. Red bars indicate CentO satellite arrays, shown in greater detail above each chromosome map. Between four and 22 cosegregated RFLP markers were anchored to each centromere. Arrows represent the locations of physical gaps on the current sequence maps. The size for four of the gaps was estimated by FISH, including the Cen4 gap [15], the first gap in Cen10 [16], the first gap in Cen7, and the second gap in Cen11 [17]; the remaining gaps were sized by optical mapping [18]. (285 KB PDF) [file pbio.0060286.sg001.pdf]

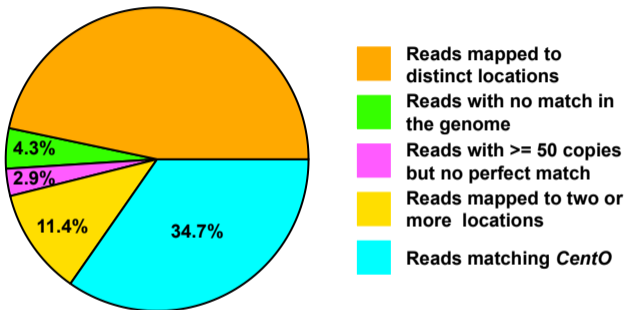

**Figure S2**

Supplement: Figure S2 — Reads showing alignments of ≥20 bp with CentO consensus were classified as CentO-containing reads. Megablast was used to map the CentO-less reads to the genome. For reads with fewer than 50 copies, the cutoff for assigning distinct mapping locations was a minimal similarity of 97% and a minimal coverage of 90%; but for those with 50 or more copies, only reads with a single perfect match were mapped. (189 KB PDF) [file pbio.0060286.sg002.pdf]

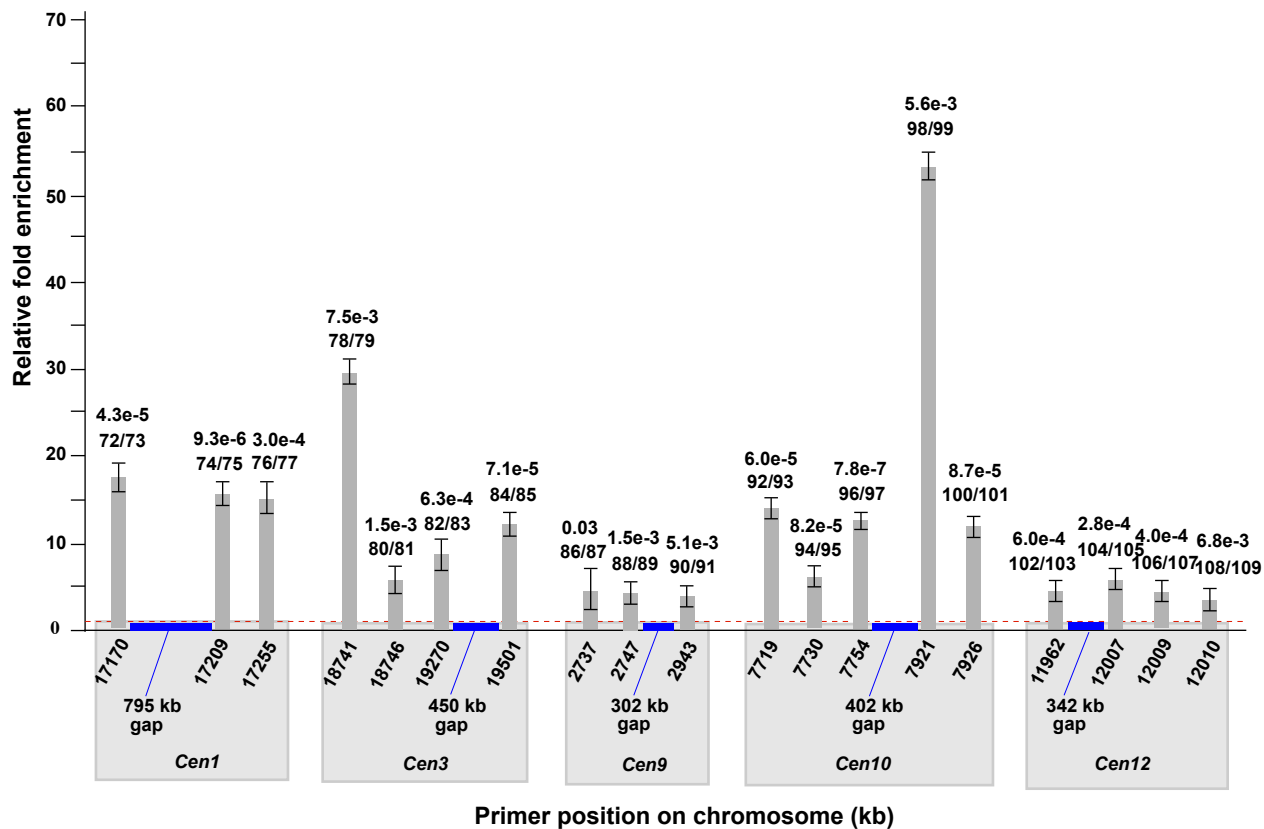

Supplement: Figure S3 — The relative positions of primers on individual chromosomes are not shown to scale. Blue bars represent physical gaps on the current sequence maps. Primer numbers are shown above each horizontal gray bar and are detailed in Table S1. Cen8.t00421.1, an active gene located approximately 605 kb away from the left boundary of the CENH3 binding domain in Cen8, was used as the negative control, whose relative fold enrichment was set at 1 and used as the baseline (dashed line). For each primer pair, significance of enrichment from antibody-binding fraction over mock treatment was tested using a one-tailed Student t-test (α = 0.01, n = 3); the corresponding p-values are shown above the primer numbers. The relative enrichment is shown as mean ± SD. (239 KB PDF) [file pbio.0060286.sg003.pdf]

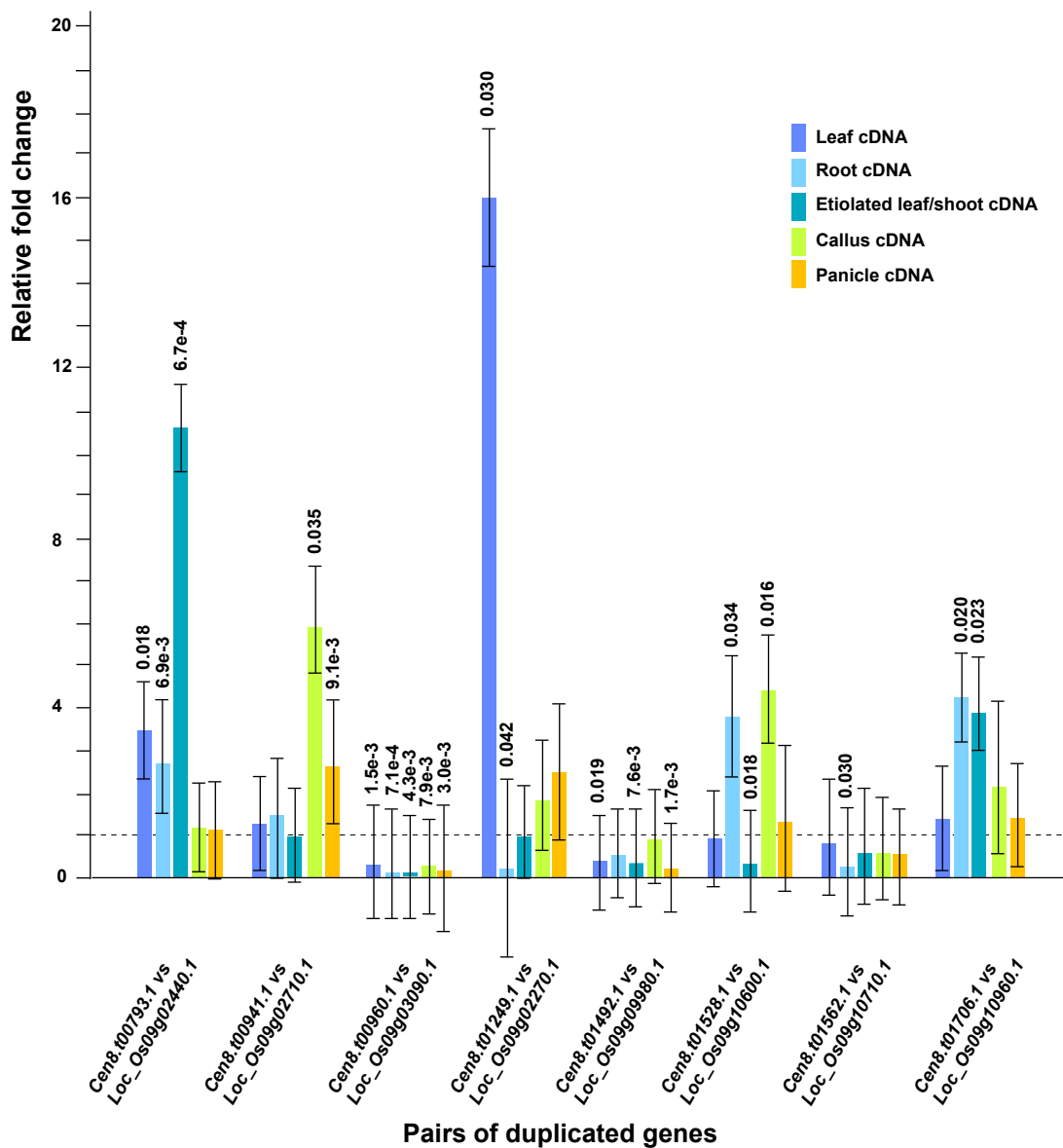

**Figure S4**

Supplement: Figure S4 — For each gene pair, relative fold change of the Cen8 copy over the chromosome 9 copy was calculated in each cDNA sample, normalized by using the real-time PCR cycle threshold (CT) difference on genomic DNA (see Materials and Methods for details). The baseline (relative fold change = 1) stands for the same level of expression between the two copies of a given gene pair. Significance of up- or down-regulated expression was inferred by a two-tailed Student t-test (α = 0.05, n = 2). The p-values of <0.05 are shown above each bar. (214 KB PDF) [file pbio.0060286.sg004.pdf]

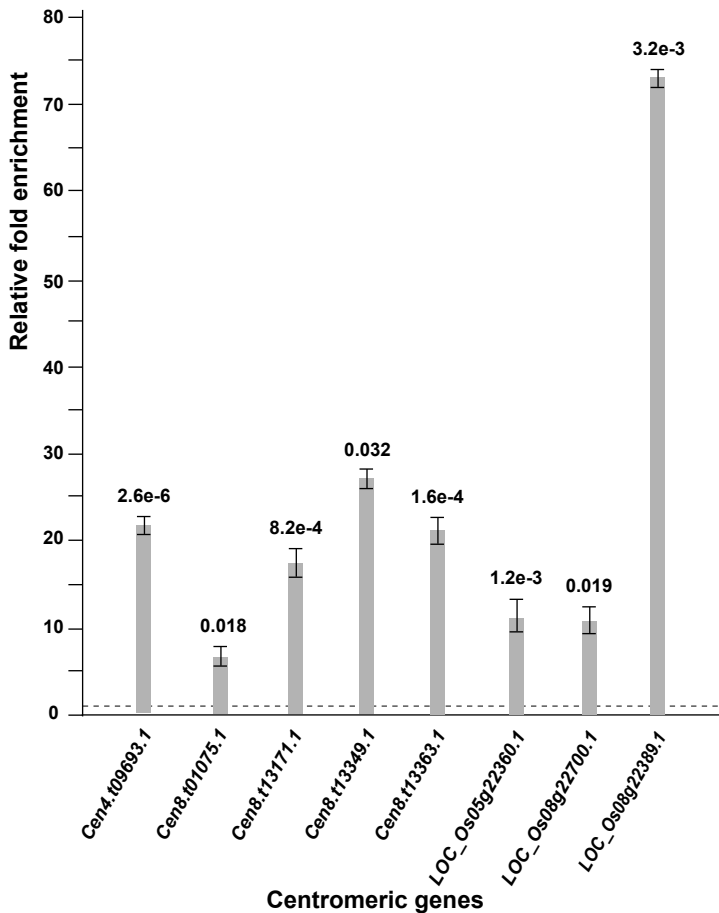

**Figure S6**

Supplement: Figure S6 — The five genes on the left side are supported by ESTs, and the other three are hypothetical genes. Relative enrichment was calculated and tested for significance as in Figure S3. Real-time PCR confirmed the CENH3 binding for all eight genes. (190 KB PDF) [file pbio.0060286.sg006.pdf]

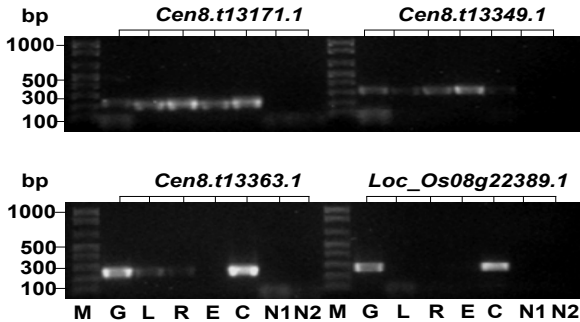

**Figure S7**

Supplement: Figure S7 — Loc_Os08g22389.1 is a hypothetical gene, whereas the other three are TE-related genes supported by unspliced ESTs. C, cDNA from calli; E, cDNA from etiolated leaves/shoots; G, genomic DNA; L, cDNA from leaves; M, molecular marker; N1, negative control (without adding reverse transcriptase) for leaves and roots; N2, negative control for etiolated leaves/shoots and calli; R, cDNA from roots. (285 KB PDF) [file pbio.0060286.sg007.pdf]
