# Supplementary material for: Intergenic Locations of Rice Centromeric Chromatin
Source: PLoS Biol. 2008 Nov 25;6(11):e286. doi: 10.1371/journal.pbio.0060286 (PMC2586382; doi:10.1371/journal.pbio.0060286)
Supplement: Figure S5 — Annotation is as described in legend to Figure 4. Two of the three ESTs that appear in gray-shaded areas correspond to 1-kb windows that are not enriched for CENH3, but that are surrounded by enriched windows. The third EST (corresponding to Cen4.t09693.1, positioned at ∼400 kb) is in a CENH3-enriched window (see Figure S6). (560 KB PDF) [file pbio.0060286.sg005.pdf]

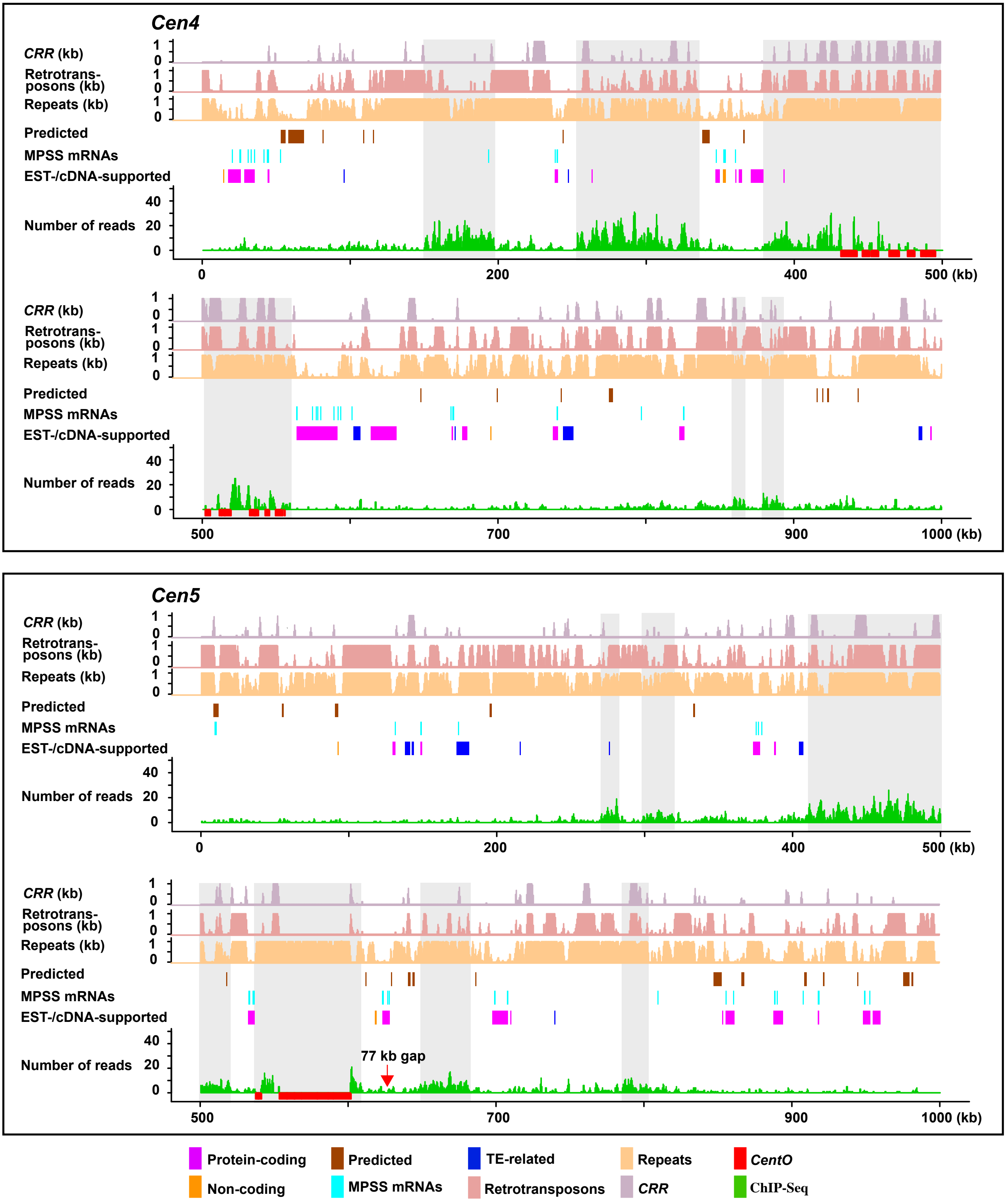

Cen4
CRR (kb)
Retrotrans-posons (kb)
Repeats (kb)
Predicted
MPSS mRNAs
EST-/cDNA-supported
Number of reads
0
200
400
500 (kb)
500
700
900
1000 (kb)
Cen5
0
200
400
500 (kb)
77 kb gap
500
700
900
1000 (kb)
Protein-coding
Non-coding
Predicted
MPSS mRNAs
TE-related
Retrotransposons
Repeats
CRR
CentO
ChIP-Seq

Figure S5
