# Supplementary material for: Intergenic Locations of Rice Centromeric Chromatin
Source: PLoS Biol. 2008 Nov 25;6(11):e286. doi: 10.1371/journal.pbio.0060286 (PMC2586382; doi:10.1371/journal.pbio.0060286)
Supplement: Table S1 — (88 KB PDF) [file pbio.0060286.st001.pdf]

Table S1. Primers Used in the Study

Primer No.	Primer Name[a]	Sequence (5' to 3')	Chr	Chr Start (bp)	Chr End (bp)	Strand	Tm (ºC)	PCR[b]
1	*Cen4.t09693.1*F1	ATGCTCATCGGGTGGTTTGATTT	4	9693110	9693132	minus	64.6	A, C
2	*Cen4.t09693.1*R1	CCACGATCAACATCTGACCTATGAAG	4	9692742	9692767	plus	64	A
3	*Cen4.t09693.1*R2	ATATGGAGCACGATCTGCATTGTCA	4	9692928	9692952	plus	65.6	C
4	*Cen8.t13171.1*F1	CCATTTTGTTGTGCTGATCCTCCT	8	13171179	13171202	minus	64.4	A, C
5	*Cen8.t13171.1*R1	GGGCAACAGGACAGTAACTGAGTACC	8	13170991	13171016	plus	64.6	A
6	*Cen8.t13171.1*R2	CAATCACATGGCTACCCGTTCG	8	13170862	13170883	plus	65	C
7	*Cen8.t13349.1*F1	TGACTTTTCATAATGCCACGCAAG	8	13355941	13355964	plus	64.3	A, C
8	*Cen8.t13349.1*R1	ACTTGGAAGAGCCTGGAATATGGTCT	8	13356222	13356247	minus	65.2	A, C
9	*Cen8.t13363.1*F1	CGTGGGTGGAAATCAGCAAACTAT	8	13362928	13362951	plus	64.4	A, C
10	*Cen8.t13363.1*R1	TACCAACAAGAACATCCGGTGCA	8	13363159	13363181	minus	65	A, C
11	*Cen8.t01075.1*F2	CCCACATTTGATGCTAACATAACTAACTAAC	8	13468687	13468717	plus	64.2	C
12	*Cen8.t01075.1*R2	CACATGACTGAGTCGTCGGGAAC	8	13468912	13468934	minus	64.1	C
13	*LOC_Os05g22360.1*F1	AGTTGGGAGCGACCAAAAGAGAAT	5	12587158	12587181	minus	64.7	A
14	*LOC_Os05g22360.1*R1	TTACTCATACATGATTTTGAATGCATAA	5	12586461	12586488	plus	60.1	A
15	*LOC_Os05g22360.1*F2	ATCTCCTTTGGAGCCTTGTTCAAAT	5	12586760	12586784	plus	64	C
16	*LOC_Os05g22360.1*R2	GAGACTGATTTAGCTTAAGAGGGTGCAT	5	12587010	12587037	minus	64.6	C
17	*LOC_Os08g22389.1*F1	GCTTTTTAAGGAGCTCCATGTCTAACTTC	8	13372773	13372801	plus	65.5	A, C
18	*LOC_Os08g22389.1*R1	TCAGAGAAAAGCATTTTGTGAATTTCTTA	8	13373007	13373035	minus	64	A, C
19	*LOC_Os08g22700.1*F1	GATGATGGCCAGCACTTCGTCTT	8	13520177	13520199	plus	65.3	A, C
20	*LOC_Os08g22700.1*R1	ACGTCCAGAACGTCGTCATCCTC	8	13520852	13520874	minus	65	A
21	*LOC_Os08g22700.1*R2	GGTCTAAAAAACGTCATAAAATATCTGATGG	8	13520375	13520405	minus	65.1	C
22	*LOC_Os02g28600.1*F1	CCACAGAATTGACAGTAGCAAGCGTC	2	16919285	16919310	plus	66.5	A
23	*LOC_Os02g28600.1*R1	CATTCTTGCGTGATGGATCATGTG	2	16919404	16919427	minus	64.9	A
24	*LOC_Os08g29910.1*F1	CAGGCCTCTTGCATCAGGTATTCT	8	18245350	18245373	plus	63.9	A
25	*LOC_Os08g29910.1*R1	AACTTGCCATGCTAAGTGCGC	8	18246434	18246454	minus	62.9	A
26	*LOC_Os08g23570.1*F1	GTTCTGTTGCTGTGGATGATTATCTGG	8	14128866	14128892	minus	65.4	A
27	*LOC_Os08g23570.1*R1	GCGTGGGCATAGCTTGAGTGC	8	14128482	14128502	plus	65.3	A
28	*LOC_Os09g09460.1*F3	CAACTCCAACGACCTGACGCTC	9	5090004	5090025	minus	64.3	A
29	*LOC_Os09g09460.1*R3	CACGTTCTTGAACCCCTGGTAGAA	9	5089851	5089874	plus	64.5	A
30	*Cen4.t09695.1*ψF1	ACTAGGGTACGACGGCGCAAAT	4	9694505	9694526	plus	64.9	A
31	*Cen4.t09695.1*ψR1	CGTCACTGTCGACCACGACCA	4	9694636	9694656	minus	64.7	A
32	*Cen7.t12104.1*ψF1	CATCGCTCGGGAACACACTTG	7	12103765	12103785	plus	63.6	A
33	*Cen7.t12104.1*ψR1	TTCAGTCCTCGAAATACCACAGGTT	7	12104968	12104992	minus	63.4	A
34	*Cen7.t12284.1*ψF1	CATCGGAGTTCCACTGGCCATATC	7	12283821	12283844	plus	66.6	A
35	*Cen7.t12284.1*ψR1	CGTAGCCATCTGGGTCCACATCA	7	12285359	12285381	minus	66.6	A
36	*Cen8.t13108.1*ψF1	CGAGACGACCACAGGGAGGTGA	8	13108502	13108523	plus	66.7	A
37	*Cen8.t13108.1*ψR1	GTCGGCCGTCGTTTTTGGTC	8	13108674	13108693	minus	64.9	A
38	*Cen8.t13376.1*ψF1	CAGTGCTCCAATGGTGGAACG	8	13376169	13376192	minus	63.5	A
39	*Cen8.t13376.1*ψR1	GGTCGTGGCTAAGCTTCTGCTATC	8	13376054	13376074	plus	63.9	A
40	*Cen8.t00793.1*F3	TTGATAAGCACCCACAGTTCAGAAAGT	8	12960403	12960429	minus	64.9	B
41	*Cen8.t00793.1*R3	TTCGCTTGAAATGTTCTCGTATAAATCTC	8	12960271	12960299	plus	65.2	B
42	*LOC_Os09g02440.1*F3	TTGGTAAGCACCCTGATTTCAGGA	9	1006208	1006231	minus	64.9	B

43	*LOC_Os09g02440.1*R3	CGGGTGAGATGATTCTGTATAAAATCG	9	1006075	1006101	plus	64.7	B
44	*Cen8.t00941.1*F4	TCCTTGAAACTAAGTACCGCAGGTTA	8	13234373	13234398	plus	63.2	B
45	*Cen8.t00941.1*R3	CAAAGTTCACCTTCAAACTTTTTCTTAATT	8	13234550	13234579	minus	63.5	B
46	*LOC_Os09g02710.1*F4	TGCTACTTGAAACCAAATATCGCAGA	9	1220692	1220717	minus	64.6	B
47	*LOC_Os09g02710.1*R3	TAAGCAGTTTCTTCAGCTGTCTTCTTTATT	9	1220511	1220540	plus	64.1	B
48	*Cen8.t00960.1*F3	CTTAAGGGGCAAATGGAATCGC	8	13260990	13261011	minus	64.9	B
49	*Cen8.t00960.1*R3	GGTTTCTAACTTGTTCTTGGAACTCAGC	8	13260858	13260885	plus	64.8	B
50	*LOC_Os09g03090.1*F3	CTCTGTGTATAAGGAACAGTTTGAAGCAA	9	1463974	1464002	minus	64.2	B
51	*LOC_Os09g03090.1*R3	CCAGGCTTCTAATTTGCTCTTGGT	9	1463836	1463859	plus	63.4	B
52	*Cen8.t01249.1*F3	GATATCATTCTTGTCGAGGGCCTC	8	13780684	13780707	minus	63.2	B
53	*Cen8.t01249.1*R3	TCACTTTATAAGGATGCATCTGATTGG	8	13780582	13780608	plus	63.3	B
54	*LOC_Os09g02270.1*F3	GTCATCCTTGTGGAAGCCTTACATTTA	9	919988	920014	minus	64.5	B
55	*LOC_Os09g02270.1*R3	TACTTGATGAGAATACATCTTGCCGG	9	919893	919918	plus	63.9	B
56	*Cen8_t01492.1*F3	CAGTTGTGGATGGCCCTAATGTG	8	14220552	14220574	plus	64.1	B
57	*Cen8_t01492.1*R3	GAAATTGCCAGGTAATGTGCTCTTC	8	14220826	14220850	minus	63.6	B
58	*LOC_Os09g09980.1*F3	CCGGTTGTAGATGGTCCTAACACA	9	5431231	5431254	plus	63	B
59	*LOC_Os09g09980.1*R3	TCTCAAAGTTTCCAGGTAGTATGCTCTTT	9	5431506	5431534	minus	64.3	B
60	*Cen8.t01528.1*F3	TGCAGCTACCGGTATGCAGATG	8	14282244	14282265	plus	63.6	B
61	*Cen8.t01528.1*R3	GAAGAACCAAGAGGGCTGGAGTAG	8	14282385	14282408	minus	62.8	B
62	*LOC_Os09g10600.1*F3	CAGTGACTACTGGCTTGCAGATGG	9	5781545	5781568	plus	63.8	B
63	*LOC_Os09g10600.1*R3	AGAGAAACAAACGGCCCTGCT	9	5781690	5781710	minus	62.9	B
64	*Cen8.t01562.1*F3	TGGGCTTCAGGCAGATATGGTG	8	14344933	14344954	plus	64.8	B
65	*Cen8.t01562.1*R3	TGCCTTTTGTACCAGGTGGAGCTA	8	14345127	14345150	minus	65.1	B
66	*LOC_Os09g10710.1*F3	AGGGCCTCAAGCAATGTTACCC	9	5840681	5840702	minus	64.3	B
67	*LOC_Os09g10710.1*R3	GCTCCTGATGAAGCTCCCTTTTTAG	9	5840499	5840523	plus	64.4	B
68	*Cen8.t01706.1*F3	TGAAGAAATTAAGCACCCTTGTACGG	8	14608399	14608424	plus	65.2	B
69	*Cen8.t01706.1*R3	AATCATGAAAGGATTGTCAGCCACAG	8	14608663	14608688	minus	65.7	B
70	*LOC_Os09g10960.1*F3	AGTTGAATGCAAGCGTGCGGTA	9	6046381	6046402	minus	65.4	B
71	*LOC_Os09g10960.1*R3	CACCATGAAGGGATTCTCAGAAACTG	9	6046119	6046144	plus	65.4	B
72	1_17170F1	AGAGTTACTCGACAAGGGTCGTGCT	1	17170240	17170264	plus	65	C
73	1_17170R1	AAATCTCCATACGAGCTCCGTTTTC	1	17170461	17170485	minus	65	C
74	1_17209F1	AGGGCGTCCTCCCTCTGTTTAGAGT	1	17208962	17208986	plus	67.1	C
75	1_17209R1	GGCCCACGAGTACTTGATAGAAAGC	1	17209145	17209169	minus	64.9	C
76	1_17255F1	TGCATTCTGGATACGCTCTTCTCTC	1	17255541	17255565	plus	64.2	C
77	1_17255R1	CCAGTAGCGGAAAGATGAGTTGCT	1	17255810	17255833	minus	64.1	C
78	3_18741F1	CGACCGTATCACCTAACCTAGTCTGA	3	18740551	18740576	plus	63.3	C
79	3_18741R1	GAAAAAGTAAATCAAAGATCCATCTACTCC	3	18740798	18740827	minus	62.9	C
80	3_18746F1	ACGGCCTTTTTGGTGATTTCTCTC	3	18745855	18745878	plus	65.1	C
81	3_18746R1	CCAAAGAGTTCAGCGCTAGATTCAA	3	18746143	18746167	minus	64.4	C
82	3_19270F1	GAAGTCAAATCCAAATAAAATAGGCGA	3	19269862	19269888	plus	64	C
83	3_19270R1	TCGAAGGGCATGCGTCTGAGTAAT	3	19270102	19270125	minus	67.1	C
84	3_19501F1	GCATGTGTGTGGATTGTAGAGCAA	3	19501386	19501409	plus	62.6	C
85	3_19501R1	TTAATCTCATGAAAGTACTGGGTGCAT	3	19501617	19501643	minus	62.9	C
86	9_02737F1	GCCTAATTTCAAGTCCGTATGACAAAG	9	2737139	2737165	plus	64.3	C
87	9_02737R1	TAGAGTTTCGAGTTCTTGCCACCAC	9	2737354	2737378	minus	64	C

88	9_02747F1	TCAACGGAGTAGGAAACCGTCAGT	9	2747689	2747712	plus	64.1	C
89	9_02747R1	ATCGAGCAGGTGATTGTGGGACA	9	2747927	2747949	minus	66.2	C
90	9_02943F1	CAAAACTCCTTTGGTTTCTGATGGA	9	2942575	2942599	plus	63.8	C
91	9_02943R1	TGATGTTGAGGAAGAGTCGGCTAAG	9	2942830	2942854	minus	64.1	C
92	10_07719F1	TGGAAACGGCTAAGTCAAGTTGTTC	10	7719312	7719336	plus	64.1	C
93	10_07719R1	TAGCCTGAGTTGCCTGTAATCGTTC	10	7719499	7719523	minus	64.2	C
94	10_07730F1	ACGAATGAGAACCATGGCCGAAG	10	7729792	7729814	plus	67.2	C
95	10_07730R1	CCACCGCTGCTTCTTCCACTACTC	10	7729976	7729999	minus	66.4	C
96	10_07754F1	CTCCACGACGCCCTCAAAATAATAA	10	7753544	7753568	plus	65.9	C
97	10_07754R1	CATCAGTCCTTGATTCAATGGCAAG	10	7753739	7753763	minus	64.9	C
98	10_07921F1	GTTGCCGATAGTTTTTGTGAGTTGC	10	7921793	7921817	plus	64.4	C
99	10_07921R1	CACAGAAAATCTTATCTCAAACGGACAA	10	7922089	7922116	minus	64.6	C
100	10_07926F1	CCGACTCCTAAAACGGTCTCACAAG	10	7925537	7925561	plus	65.6	C
101	10_07926R1	TCTTATTAAAATCGGGAAGTTGGAGGA	10	7925730	7925756	minus	65.3	C
102	12_11962F1	GCATGGGGTACCCAAGAAGATTGTA	12	11962176	11962200	plus	65.5	C
103	12_11962R1	GGCTTGGTAGCTGTTGTTGTACGAG	12	11962404	11962428	minus	64.5	C
104	12_12007F1	GGACATCAACACCATCGATACATCC	12	12007093	12007117	plus	64.3	C
105	12_12007R1	AAATCGGAGTTCGCATGAAGTCGT	12	12007339	12007362	minus	66.2	C
106	12_12009F1	TTTAGCCATTGCTACTTGCTTGCAG	12	12008614	12008638	plus	65.3	C
107	12_12009R1	CCGACTACAACCCTACAAGACGTTG	12	12008839	12008863	minus	64.3	C
108	12_12010F1	TCAGAGTTGTGCATGAGTTGTTCCT	12	12009951	12009975	plus	62.9	C
109	12_12010R1	GATGTACACCGACTGAACCAAGCAC	12	12010204	12010228	minus	64.8	C

[a]F1 to F4: forward primer; R1 to R4: reverse primer.
[b]A: RT-PCR; B: real-time RT-PCR; C: ChIP real-time PCR.
