# Supplementary material for: Intergenic Locations of Rice Centromeric Chromatin
Source: PLoS Biol. 2008 Nov 25;6(11):e286. doi: 10.1371/journal.pbio.0060286 (PMC2586382; doi:10.1371/journal.pbio.0060286)
Supplement: Table S2 — (41 KB PDF) [file pbio.0060286.st002.pdf]

Table S2. Forty-three Regions from *Cen8* that Matched Active Genes[a] Elsewhere in the Genome

Category	Total Regions	Deterioration[b]
Intergenic	15[c]	13[c]
Active gene[a], exon	17	1
Best match	12	0
Not best match	5	1
Active gene[a], intron	2	2
Inactive gene, exon	6	6
Non-coding	3	3
Total	43[c]	25[c]

[a]Active genes are either supported by ESTs/cDNAs or were verified by RT-PCR.
[b]Deterioration: the presence of premature stop codon, frame shift, or truncation of CDS.
[c]Three of these regions were in the CENH3 subdomains.
