# Supplementary material for: Intergenic Locations of Rice Centromeric Chromatin
Source: PLoS Biol. 2008 Nov 25;6(11):e286. doi: 10.1371/journal.pbio.0060286 (PMC2586382; doi:10.1371/journal.pbio.0060286)
Supplement: Table S3 — (66 KB PDF) [file pbio.0060286.st003.pdf]

Table S3. Fourteen Pairs of Duplicated Genes in Nipponbare and Their Respective Orthologs in Two Wild Rice Species

***Cen8* gene**							***Chr9* paralog**						
**Name**	**Genomic DNA size (bp)**	**Protein length (aa)**	***O. brachyantha* GSS accession**	**Protein identity (%)**	***O. granulata* GSS accession**	**Protein identity (%)**	**Name**	**Genomic DNA size (bp)**	**Protein length (aa)**	***O. brachyantha* GSS accession**	**Protein identity (%)**	***O. granulata* GSS accession**	**Protein identity (%)**
Cen8.t00793.1	4612	192	-				*LOC_Os09g02440.1*	5755	159				
Cen8.t00941.1	8518	235					*LOC_Os09g02710.1*	3691	227	CL547383.1	89		
Cen8.t00960.1	5945	279					*LOC_Os09g03090.1*	6115	269				
Cen8.t01003.1	7461	660					*LOC_Os09g02700.1*	5156	662			DU818528.1	95
Cen8.t01249.1	6852	270					*LOC_Os09g02270.1*	5451	267				
Cen8.t01272.1	3911	384	CL583551.1	78			*LOC_Os09g08130.2*	3349	381				
Cen8.t01289.1	3051	415			CZ155639.1	92	*LOC_Os09g07350.1*	3718	401	EI043679.1	98		
Cen8.t01413.1	901	150					*LOC_Os09g09460.1*	1469	339	CL543704.1	90		
Cen8.t01492.1	5235	499					*LOC_Os09g09980.1*	2340	488	CL572619.1, CL574365.1, CL541635.1	95-97	DU656519.1, DX119853.1	97
Cen8.t01528.1	4656	375			DU650525.1	88	*LOC_Os09g10600.1*	4201	371	CL576330.1	85	DX120313.1	96.7
Cen8.t01548.1	824	150	CL547680.1	76			*LOC_Os09g10620.1*	558	185				
Cen8.t01562.1	6365	458	CL571360.2	90	DU652035.1	95	*LOC_Os09g10710.1*	7239	409	EI046664.1, EI046356.1	84-91		
Cen8.t01706.1	20851	1657	CL561448.1, CL597792.1	92-96			*LOC_Os09g10960.1*	11395	1740			DU156458.1, DU816804.1	93-100
Cen8.t01718.1	5977	481	CL589336.1	79			*LOC_Os09g10950.1*	4043	440				

Orthologs were identified by Tblastn against *O. brachyantha* and *O. granulata* genome survey sequences (GSS) available in GenBank.
