# Supplementary material for: Intergenic Locations of Rice Centromeric Chromatin
Source: PLoS Biol. 2008 Nov 25;6(11):e286. doi: 10.1371/journal.pbio.0060286 (PMC2586382; doi:10.1371/journal.pbio.0060286)
Supplement: Table S4 — (72 KB PDF) [file pbio.0060286.st004.pdf]

Table S4. Active Genes Identified from Three 1-Mb Sequences Spanning the CENH3 Binding Domain of *Cen4*, *Cen5* and *Cen7*

Gene no.	Gene model[a]	Chr start (bp)	Chr end (bp)	Strand	Representative transcripts	Protein
1	*Cen4.t09317.1*	9316614	9325127	plus	AK103892	Electron carrier/ protein disulfide oxidoreductase
1	*Cen4.t09317.2*	9316614	9325127	plus	CT861680	Electron carrier/ protein disulfide oxidoreductase
1	*Cen4.t09317.3*	9316614	9325127	plus	CB685658	Electron carrier/ protein disulfide oxidoreductase
1	*Cen4.t09317.4*	9316614	9325127	plus	CI263084	Electron carrier/ protein disulfide oxidoreductase
2	*Cen4.t09327.1*	9327475	9334597	plus	AK065922	Expressed protein
2	*Cen4.t09327.2*	9327475	9334597	plus	CI764313	Expressed protein
3	*Cen4.t09343.1*	9343209	9344399	minus	CA754130, AK121488, CK009863	Metal ion binding protein
4	*Cen4.t09538.1*	9537710	9539977	minus	AK066605, CB685856	Expressed protein
4	*Cen4.t09538.2*	9537710	9539977	minus	AK100644, CI312953, CB684892	Expressed protein
4	*Cen4.t09538.3*	9537710	9539977	minus	CI240242, AU029394	Expressed protein
5	*Cen4.t09563.1*	9562846	9563252	minus	CI624175	Expressed protein
6	*Cen4.t09646.1*	9646495	9649413	minus	AK069432, CI363224, CF333914	Senescence-associated family protein
6	*Cen4.t09646.2*	9646495	9649413	minus	CF332905, CI263772	Senescence-associated family protein
6	*Cen4.t09646.3*	9646495	9649413	minus	CF336068, CF332906	Senescence-associated family protein
7	*Cen4.t09660.1*	9659838	9660402	plus	CI712709, CI510192	Expressed protein
8	*Cen4.t09662.1*	9662268	9664470	plus	AK120707	Expressed protein
8	*Cen4.t09662.2*	9662268	9664470	plus	AK058584	Expressed protein
8	*Cen4.t09662.3*	9662268	9664470	plus	CI491886, CI018118	Expressed protein
9	*Cen4.t09670.1*	9670366	9678831	plus	AK066975, CI567737	Kinesin heavy chain isolog
10	*Cen4.t09693.1*	9692587	9693144	minus	CK035758	Expressed protein
11	*Cen4.t09864.1*	9864042	9891552	minus	AK121105, AK120441, CB627180	Cleavage and polyadenylation specificity factor (CPSF)
11	*Cen4.t09864.2*	9864042	9891552	minus	AK072371	Cleavage and polyadenylation specificity factor (CPSF)
12	*Cen4.t09914.1*	9914135	9931621	minus	AK119369	Protein transporter KEU (KEULE)
12	*Cen4.t09914.2*	9914135	9931621	minus	CB096998	Protein transporter KEU (KEULE)
12	*Cen4.t09914.3*	9914135	9931621	minus	CT846694, CK045115	Protein transporter KEU (KEULE)
13	*Cen4.t09969.1*	9968766	9969779	plus	AK073668	Putative histone H1 protein
13	*Cen4.t09969.2*	9968766	9969779	plus	CT845176	Putative histone H1 protein
14	*Cen4.t09976.1*	9975709	9979187	minus	AK072363	Expressed protein
15	*Cen4.t10037.1*	10037029	10040474	plus	AK071042	Dihydrodipicolinate synthase
16	*Cen4.t10122.1*	10122442	10125948	plus	CB672251, CI677244, CB672252, CI225308	Cytochrome P450 like protein
17	*Cen4.t10292.1*	10291999	10292880	minus	AK107745	Putative AP2/EREBP transcription factor
18	*Cen5.t12030.1*	12030187	12032043	plus	CI515952	Expressed protein
19	*Cen5.t12049.1*	12048811	12049869	plus	AK120717	Expressed protein, Invertase/pectin methylesterase inhibitor family protein

20	*Cen5.t12273.1*	12273223	12277825	plus	AK073229	Expressed protein
20	*Cen5.t12273.2*	12273223	12277825	plus	AK061533	Expressed protein
21	*Cen5.t12287.1*	12287360	12288593	plus	AK108829, CT843968	Zinc finger (C2H2 type) family protein
22	*Cen5.t12433.1*	12432546	12437099	plus	AK071727, CI669617	Expressed protein
22	*Cen5.t12433.2*	12432546	12437099	plus	CB623020	Expressed protein
22	*Cen5.t12433.3*	12432546	12437099	plus	CB659867	Expressed protein
22	*Cen5.t12433.4*	12432546	12437099	plus	CI657923, CT843899, CB670241	Expressed protein
23	*Cen5.t12523.1*	12523423	12528409	minus	AK100478	Crooked neck protein, putative
23	*Cen5.t12523.2*	12523423	12528409	minus	CB675142, CV720930	Crooked neck protein, putative
24	*Cen5.t12597.1*	12597773	12608441	minus	CB633315, CB636241, CK056833	N-acetylglucosaminyl transferase component family protein
24	*Cen5.t12597.2*	12597773	12608441	minus	CB645379	N-acetylglucosaminyl transferase component family protein
24	*Cen5.t12597.3*	12597773	12608441	minus	CF304987, CA764504	N-acetylglucosaminyl transferase component family protein
25	*Cen5.t12610.1*	12610056	12610650	plus	BX900003	Expressed protein
26	*Cen5.t12753.1*	12753414	12754077	plus	AK073226	Expressed protein
27	*Cen5.t12756.1*	12755759	12761715	minus	AK073234	Expressed protein
27	*Cen5.t12756.2*	12755759	12761715	minus	AK099865	Expressed protein
27	*Cen5.t12756.3*	12755759	12761715	minus	CB626539	Expressed protein
28	*Cen5.t12788.1*	12788199	12794695	plus	AK065373	Transcription factor-related protein
29	*Cen5.t12818.1*	12818196	12819118	plus	AK059562, CI698477	One helix protein OHP
30	*Cen5.t12849.1*	12848728	12853614	minus	CF988794, CI386100, CI474855	Expressed protein
31	*Cen5.t12855.1*	12855388	12860663	minus	AK072592	Pectate lyase family protein
32	*Cen7.t11614.1*	11613954	11614678	plus	CB678790	Expressed protein
33	*Cen7.t11617.1*	11617380	11619279	plus	CI680881, CA759611	Expressed protein
34	*Cen7.t11626.1*	11625713	11626690	plus	AK071440	MATH domain containing protein
35	*Cen7.t11628.1*	11627789	11628212	minus	CI422943, CI745319	Expressed protein
36	*Cen7.t11630.1*	11630149	11634963	minus	AU166460, CK006432, AU166459	Expressed protein
37	*Cen7.t11641.1*	11640857	11642585	plus	AK101219	Expressed protein
38	*Cen7.t11683.1*	11683053	11685569	minus	AK071774	Expressed protein
39	*Cen7.t11703.1*	11702987	11703528	plus	CI670206, CI447782	Expressed protein
40	*Cen7.t11737.1*	11737025	11739370	minus	AK064565	Metal ion binding protein
41	*Cen7.t11778.1*	11778475	11784573	plus	CT859796	Expressed protein
42	*Cen7.t11844.1*	11844119	11850166	plus	CB682996, CI622023, AK120733, CK060665, CK051454, CB682997, CI081406, CK037201	Pentatricopeptide (PPR) repeat-containing protein
43	*Cen7.t11864.1*	11863688	11872547	minus	AK061941	Lysine-sensitive aspartate kinase
43	*Cen7.t11864.2*	11863688	11872547	minus	CK065410, CK010074, CI045862, CI128231	Lysine-sensitive aspartate kinase
44	*Cen7.t11885.1*	11885428	11892727	minus	AK073463	RNA helicase

44	*Cen7.t11885.2*	11885428	11892727	minus	AK102570	RNA helicase
44	*Cen7.t11885.3*	11885428	11892727	minus	CB669398, CI647857, CI478533	RNA helicase
45	*Cen7.t12312.1*	12311799	12324095	minus	AK101785, CT845833, CI526911	Zinc finger (Ran-binding) family protein
46	*Cen7.t12422.1*	12422366	12438792	plus	AK111882, CI736281	WD-40 repeat family protein
47	*Cen7.t12543.1*	12542517	12548366	minus	AK064867, CI304968, CV733616	Glucose-6-phosphate 1-dehydrogenase
48	*Cen7.t12564.1*	12564136	12568298	plus	CK054720, CK082018, CT843869	Expressed protein
49	*Cen7.t12573.1*	12572886	12577971	minus	AK101812	DNA primase, large subunit family
49	*Cen7.t12573.2*	12572886	12577971	minus	AK064087	DNA primase, large subunit family

[a]Suffix ".1", ".2", ".3" and ".4" represent different splicing isoforms of the same gene.
