# Supplementary material for: Intergenic Locations of Rice Centromeric Chromatin
Source: PLoS Biol. 2008 Nov 25;6(11):e286. doi: 10.1371/journal.pbio.0060286 (PMC2586382; doi:10.1371/journal.pbio.0060286)
Supplement: Table S5 — (59 KB PDF) [file pbio.0060286.st005.pdf]

Table S5. Eight Pseudogenes Identified from the CENH3 ChIP-Seq Peaks

Pseudogene	Chr start (bp)	Chr end (bp)	Length (bp)	Deterioration	Expression[a]	Coverage of parent protein (%)	Parent gene	No. coding exon
Cen4.t09593.1 ψ	1222768	1222974	207	Truncated CDS	NA	49	*LOC_Os01g37280.1*	*1*
Cen4.t09695.1 ψ	1324491	1324943	453	Premature stop codon	No	50	*LOC_Os04g32240.1*	*3*
Cen7.t12104.1 ψ	12103758	12105033	1276	Premature stop codon	No	46	*LOC_Os12g37519.1*	*3*
Cen7.t12280.1 ψ	12281185	12282756	1572	Frame shift	NA	49	*LOC_Os12g44100.1*	*2*
Cen7.t12284.1 ψ	12283786	12285419	1634	Not found	No	91	*LOC_Os01g28970.2*	*2*
Cen8.t13108.1 ψ	13108381	13108698	318	Premature stop codon	No	66	*LOC_Os05g10754.1*	*1*
Cen8.t13153.1 ψ	13152862	13153173	312	Truncated CDS	NA	83	*LOC_Os09g06570.1*	*1*
Cen8.t13376.1 ψ	13375898	13376350	453	Truncated CDS	No	88	*LOC_Os01g28970.1*	*1*

[a]Five pseudogenes were tested for expression, none of them were found to be transcribed in leaves, roots, etiolated leaves/shoots and calli. NA: expression not tested.
